# Clinical and Functional Characterization of CD-NTase Enzymes in Esophageal Squamous Cell Carcinoma

**DOI:** 10.7150/jca.100226

**Published:** 2025-06-12

**Authors:** Zhiwen Gong, Hongcheng Zhong, Shijiancong Liu, Xujie Xiao, Yuanquan Wu, Xiaojian Li, Qingdong Cao

**Affiliations:** 1 Department of Thoracic Surgery, The Fifth Affiliated Hospital, Sun Yat-sen Unversity, Zhuhai 519000, China.; 2 Guangdong-Hong Kong-Macao University Joint Laboratory of Interventional Medicine, the Fifth Affiliated Hospital of Sun Yat-Sen University, Zhuhai 519000, China.; 3 Department of Gastrointestinal Surgery, The Affiliated Kashi Hospital, Sun Yat-sen University, Kashi 844099, China.; 4 Department of Gastrointestinal Surgery, The First People's Hospital of Kashi Prefecture, Kashi 844099, China.

**Keywords:** CD-NTase enzymes, MB21D2, OS, biological function, Wnt/β-catenin signaling pathway

## Abstract

**Purpose:** The cGAS/DncV-like nucleotidyltransferase (CD-NTase) enzyme family plays a critical role in tumor development, but its clinical significance and biological function in esophageal squamous cell carcinoma (ESCC) remain unclear.

**Methods:** We analyzed 352 ESCC cases, including 260 from public datasets (TCGA, GSE53624, and GSE53622) and 92 from our clinical cohort. Candidate CD-NTase enzymes were validated through *in vivo* and *in vitro* experiments.

**Results:** Analysis of 11 CD-NTase enzymes identified MB21D2 as the only significant prognostic factor in three clinical cohorts. Patients with low MB21D2 expression demonstrated markedly worse overall survival (OS). Multivariate analysis indicated that low MB21D2 was an independent prognostic factor (HR = 2.5, P =0.04; HR = 1.33, P =0.02; HR = 2.5, P =0.02). Furthermore, biological functional experiments showed that knockdown MB21D2 promotes proliferation, migration, and invasion in ESCC cells. While overexpression MB21D2 has the opposite effect. RNA-seq and western blotting analysis revealed that knockdown of MB21D2 activates markers associated with the Wnt/β-catenin signaling pathway, thereby promoting ESCC progression.

**Conclusion:** MB21D2 serves as a critical prognostic and functional factor in ESCC progression, offering a potential therapeutic target for improving patient outcomes.

## Introduction

Esophageal cancer (EC) remains a global health challenge, ranking as the sixth leading cause of cancer death with over 600,000 new cases and 540,000 deaths reported in 2020 1. The two main histological types are esophageal adenocarcinoma (EADC) and esophageal squamous cell carcinoma (ESCC) 2. Histologically, squamous cell carcinoma accounts for over 85% of cases. China experiences a high incidence of EC, mostly ESCC 3, with both incidence and mortality rates among the highest globally. Despite advancements in understanding its molecular basis, the five-year survival rate remains below 20% 4,5. Recent years have seen some progress in treatment options, including new chemotherapy and radiotherapy regimens, yet the prognosis remains poor. Therefore, identifying new therapeutic targets and developing innovative treatment approaches are imperative to improve outcomes for patients with ESCC.

Neoadjuvant chemoradiotherapy followed by surgical resection is widely accepted as the standard treatment for locally advanced EC 6. However, postoperative recurrence remains common, with over 70% of patients failing to achieve pathological complete response (pCR) 7. Patients who do not reach pCR and present with positive lymph nodes have shorter survival and poorer prognosis 8,9. In the dynamic field of immunotherapy research, the Checkmate-577 trial has shown that nivolumab is an optimal adjuvant therapy for patients with non-metastatic ESCC and residual disease following multimodal treatment 10,11. However, the optimal indications and treatment regimens continue to be debated globally. This highlights the necessity of developing reliable prognostic biomarkers for ESCC, with the expectation that these biomarkers provide greater prognostic clinical efficacy than traditional clinicopathological factors.

The discovery of the cGAS/DncV-like nucleotidyltransferase (CD-NTase) family, comprising 11 members (Table S1), provides a novel perspective for understanding the molecular mechanisms underlying tumorigenesis and progression. They catalyze the synthesis of cyclic oligonucleotide signals, activating downstream effectors and thereby triggering cell death, a process crucial for tumor immune surveillance and response. In oncology, members of the CD-NTase have been shown to play a significant role in regulating the cGAS-STING signaling pathway 12. This finding highlights the potential role of the CD-NTase enzymes in modulating host antiviral and antitumor immune responses. cGAS, a crucial member, serves as a cytoplasmic DNA sensor involved in tumor immune surveillance 13, influencing both the DNA damage response and the tumor microenvironment 14,15. Furthermore, activation of the cGAS-STING pathway enhances antitumor immunity, suggesting potential applications in cancer immunotherapy 13. Recent findings also suggest that CD-NTase enzymes directly regulate tumor cell survival and proliferation by modulating intracellular signaling pathways 16, offering promising targets for new anticancer drugs. Their dual role in immune regulation and tumor cell dynamics underscores the need for further research into their mechanisms and therapeutic potential in cancer prevention, diagnosis, and treatment.

Despite mounting evidence indicating its critical role in tumor biology, the specific function of CD-NTase enzymes in human cancers, especially in the progression of ESCC tumors, has not been fully elucidated. In this study, we have comprehensively analyzed the expression of CD-NTase enzymes in ESCC and conducted a series of systematic studies to gain a better understanding of the potential molecular functions of CD-NTase enzymes in this lethal malignancy.

## Materials and Methods

### Patients and samples

Tissue samples from 92 ESCC patients were retrospectively collected for high-throughput transcriptomic analysis of RNA expression profiles. Informed consent was obtained, and clinical data were gathered for follow-up. Patients with post-operative adenocarcinoma, prior chemoradiotherapy, or incomplete follow-up were excluded. Details of patient enrollment are provided in supplementary Table S2. The study was approved by the Medical Ethics Committee (Approval No. 2020-K06-1, 8 January 2020).

### The Cancer Genome Atlas (TCGA) and Gene Expression Omnibus (GEO) data of ESCC

The transcriptome data of 33 cancer types were sourced from the TCGA database (https://portal.gdc.cancer.gov/) and refined using high-quality prognostic datasets from a prior study 17. The TCGA-ESCA dataset included 174 transcriptomes and 185 clinical cases, from which 81 paired ESCC expression matrices and clinical prognoses were extracted. Additionally, the ESCC microarray dataset GSE53625, comprising GSE53624 and GSE53622, was obtained from the GEO database (https://www.ncbi.nlm.nih.gov/geo/), covering gene expression, pathological features, and prognostic data for 119 and 60 pairs of ESCC and adjacent non-tumor tissues, respectively. Data were processed with “R” (v4.2.2), and clinicopathological characteristics of the cohorts are detailed in Table S3.

### Bioinformatics analysis

Pan-cancer analysis was performed using the “TCGAplot” (v6.2.0) package 18,19. Survival analysis dichotomized the three clinical tumor cohorts based on the optimal MB21D2 expression cutoff, identified using “survminer” (v0.4.9). This cutoff significantly stratified cohorts by overall survival (OS) (log-rank test, P < 0.05) 20. Cox proportional hazards regression models were built with the “coxph” function in the “survival” (v3.7.0) package to analyze gene expression and prognosis. Gene Ontology (GO) and KEGG pathway analyses of DEGs between sh-MB21D2 and sh-Ctrl KYSE30 cells were conducted using “clusterProfiler” (v4.12.0), with DEGs ranked by fold change for GSEA. KEGG hallmark gene sets were sourced from the Molecular Signatures Database.

### Cell lines and culture

The ESCC cell lines (KYSE30, KYSE150, EC109, TE1, KYSE450, KYSE410, KYSE510) and the 293T cell line were obtained from SHANGHAI WHELAB BIOSCIENCE LIMITED. ESCC cells were cultured in RPMI-1640 medium (Catalogue No. C11875500BT, Thermo Fisher Scientific, Inc.), and 293T cells in DMEM (Catalogue No. C11995500BT, Thermo Fisher Scientific, Inc.), both supplemented with 10% FBS, 100 U/mL penicillin, and 100 µg/mL streptomycin. The Het-1A normal human esophageal epithelial cell line (iCell) was cultured in BEGM complete medium enriched with growth factors. All cell lines were maintained at 37 ºC with 5% CO2, and regular mycoplasma testing was performed. Details of cell lines are listed in Table S4.

### Lentivirus packaging and infection

Full-length Homo-MB21D2 cDNAs were cloned into the pCDH-CMV-MCS-EF1-copGFP-T2A-Puro vector to construct MB21D2 plasmids, verified by DNA sequencing. Empty vectors served as controls. MB21D2-specific shRNA was synthesized and inserted into the pLKO.1-U6-EF1a-copGFP-T2A-puro vector, creating pLKO.1-U6-shMB21D2 plasmids, with scramble plasmids as negative controls. Plasmids were transfected into 293T cells using the Lenti-Pac HIV Kit (Catalogue No.LT001, GeneCopoeia, Inc.) for lentivirus packaging. Target cells were infected and selected with 4 µg/mL puromycin to establish stable MB21D2 knockdown (KYSE30, KYSE150) or overexpression (KYSE450, EC109) cell lines. KYSE30 cells were also transfected with pCDH-CMV-MCS-EF1-luciferase-neo for *in vivo* imaging. All expression plasmids are detailed in Table S5, with sequences provided by Guangzhou IGE Biotechnology Ltd.

### Cell viability and cell proliferation assays

Viable cells were harvested, counted, and diluted to 1 × 10⁴/mL, with 100 µL seeded into Corning 96-well plates. Cell growth and proliferation were monitored using the IncuCyte S3 system with images captured every 24 hours, and data analyzed via integrated software. For recovery experiments, ESCC cell viability post-XAV939 (10 µM) treatment was evaluated using the Cell Counting Kit-8(CCK-8, Catalogue No. C6005, New Cell & Molecular Biotech Co., Ltd.). Cells (2 × 10³/well) were seeded in 96-well plates, followed by the addition of 10 µL CCK-8 reagent per well. Absorbance at 450 nm was measured with a spectrophotometer.

### Colony formation assays

To assess the impact of MB21D2 on proliferation capacity, 5 × 10^2^ cells from control, MB21D2 knockdown or overexpression groups were seeded in cell culture plates. The medium was replaced every three days. After 14 days of culture, the cells were washed with PBS, fixed in 4% paraformaldehyde for 20 minutes, and stained with 0.1% crystal violet for 30 minutes. Finally, the colonies were photographed and counted.

### Cell migration and invasion assays

The wound-healing assay was performed using the IncuCyte S3 system. Cells were seeded in 96-well plates, allowed to reach >90% confluency, and scratched with a pin block. Serum-free medium was used for culture, with observations over 36 hours. The cell migration assay used 24-well transwell plates with an 8 μm membrane (Corning). Cells were resuspended in RPMI 1640, adjusted to specific densities (KYSE30-5×10^5^/mL, KYSE150-2.5×10^6^/mL, EC109-1×10^6^/mL, KYSE450-2×10^6^/mL), and 200 µL of suspension was added to the upper chamber, with 700 µL of medium containing 10% FBS in the lower chamber. For invasion assays, double the cell number was seeded in chambers precoated with Matrigel (200 µg/mL). After 24 hours at 37 °C, non-migrated cells were removed, and the filters were fixed, stained with 0.1% crystal violet, and photographed for analysis.

### Western blotting

Protein levels were analyzed by western blotting. Cells were lysed on ice for 30 minutes in RIPA buffer with protease and phosphatase inhibitors. Lysates were centrifuged at 12,000G for 15 minutes at 4 ºC, and protein concentrations were measured using the BCA method. Proteins (20 µg) were separated via SDS-PAGE (10% separating, 5% stacking gel) and transferred to PVDF membranes. Membranes were blocked with 5% non-fat milk for one hour at room temperature, incubated overnight at 4 ºC with primary antibodies, and then with HRP-conjugated secondary antibodies. Signals were detected using chemiluminescent reagent. Antibodies are listed in Table S6.

### Animal experiments

The experimental protocol was approved by the Ethics Committee of the Fifth Affiliated Hospital of Sun Yat-sen University (Approval No. 00332, 14 February 2023). BALB/c Nude mice (4-6 weeks old) were obtained from Vital River Laboratory Animal Technology Co., Ltd., and housed under SPF conditions at the Guangdong Provincial Key Laboratory of Biomedical Imaging. For the ESCC xenograft model, 3 × 10⁶ sh-MB21D2 KYSE30 cells were subcutaneously injected into each mouse. Tumor size was measured every four days from week one, and mice were euthanized on day 25. Tumor volume was calculated using the formula: Volume = (Length × Width^2^)/2. For the metastasis model, 2 × 10⁶ sh-MB21D2 and sh-Ctrl KYSE30 cells expressing luciferase were injected into the tail vein. Lung metastases were analyzed via IVIS Lumina III imaging based on fluorescence intensity.

### RNA-Sequencing analysis

The RNA-seq library were prepared following the standard Illumina protocol. Total RNA from sh-MB21D2 and sh-Ctrl KYSE30 cells was isolated using TRIzol reagent. The library construction, sequencing, and quality control processes were undertaken by NOVOGENE. Clean Reads were accurately aligned to the reference genome using HISAT2, and read counts for each gene were systematically tabulated. Differential expression analysis utilized the “DESeq2” (version 1.44.0) package. RNA-seq libraries from sh-MB21D2 and sh-Ctrl KYSE30 cells were prepared and sequenced by NOVOGENE. DEGs were identified using “DESeq2” (v1.44.0).

### Statistical analysis

Data were presented as means ± standard deviations (S.D) or means ± standard error of the mean (S.E.M). Comparisons between the two groups were conducted using an unpaired, two-tailed Student's t-test. The association between MB21D2 expression levels and clinicopathological parameters were assessed with the Chi-square test. All statistical analyses were conducted using Prism 9.5.1 software (GraphPad). P < 0.05 was considered to indicate statistical significance.

## Results

### Pan-cancer analysis of CD-NTase gene set in the TCGA database: Expression Patterns, Prognostic Roles and Immune Cell Infiltration

**Figure 1:**
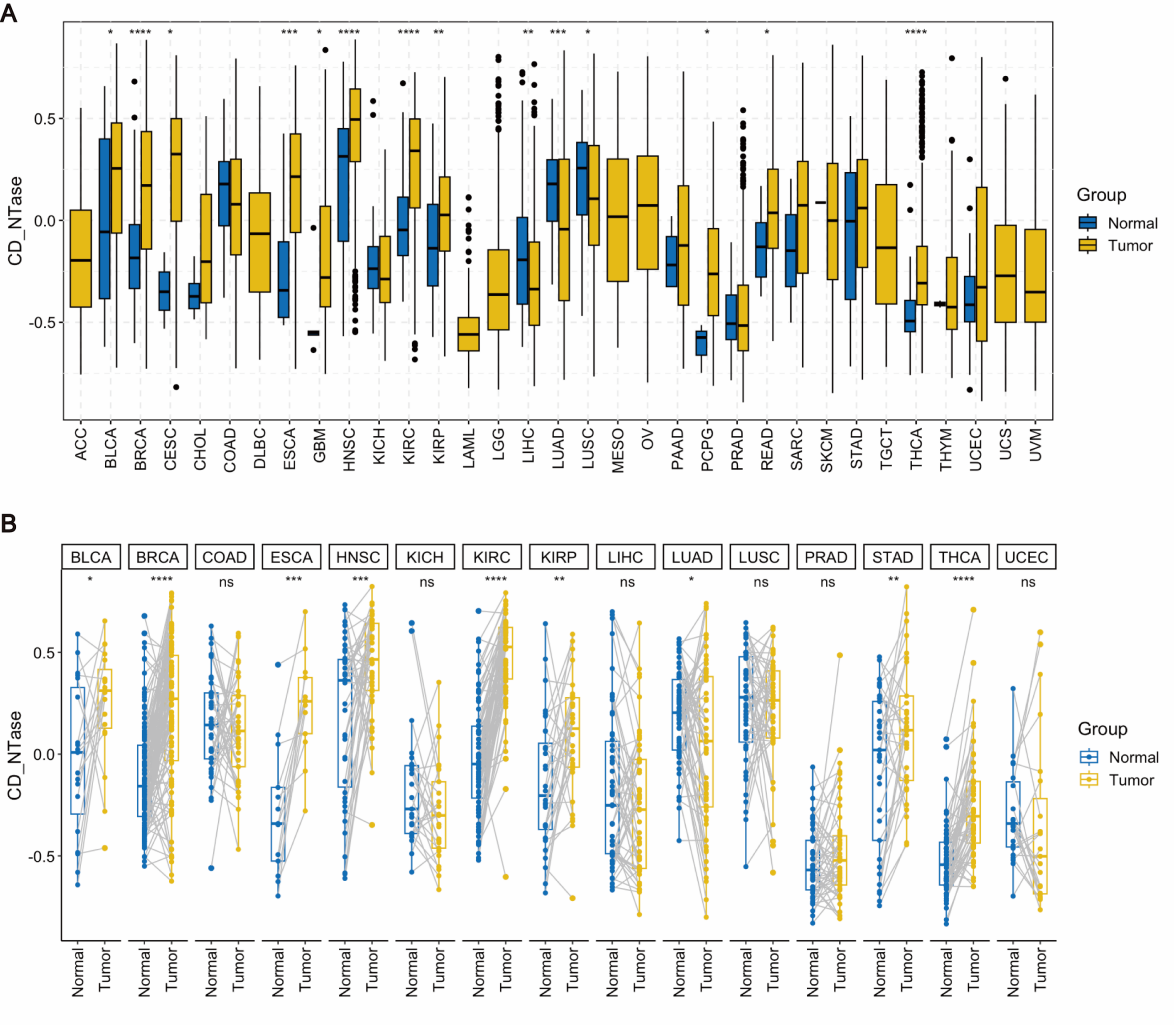
Pan-cancer expression analysis. **A** Pan-cancer expression analysis of the CD-NTase gene set score was conducted across 33 cancer types based on the TCGA database, employing the Wilcoxon test as the statistical method. **B** For 15 cancer types in the TCGA dataset with more than 20 paired samples, pan-cancer expression analysis of the CD-NTase gene set was performed using the Wilcoxon test. Tumor groups exhibiting significant diferences were marked accordingly. *P < 0.05, **P < 0.01, ***P < 0.001, ****P < 0.0001.

We performed pan-cancer expression analysis of the gene set comprising 11 CD-NTase enzymes and scored them using the 'GSVA' function. To compare the expression levels of the CD-NTase gene set between normal human tissues and cancerous tissues, we utilized the unpaired expression TPM matrix for 33 tumors from the TCGA database. Our analysis revealed that the CD-NTase gene set scores exhibit significant variations across 17 cancer types. Notably, these scores were elevated in 14 cancer types, including BLCA, BRCA, CESC, ESCA, GBM, HNSC, KIRC, KIPR, PCPG, READ, and THCA. Conversely, lower scores were observed in LIHC, LUAD, and LUSE (Fig. 1A). Further differential analysis on 15 paired cancers in TCGA showed that the CD-NTase gene set scores were significantly higher in BLCA, BRCA, ESCA, HNSC, KIRC, KIRP, STAD, and THCA compared to normal tissues, whereas the opposite was true for LUAD (Fig. 1B). Additionally, we evaluated the prognostic value of the CD-NTase gene set in various cancers. Univariate Cox analysis based on overall survival (OS) for 33 cancers indicated that the CD-NTase gene set acted as a protective factor against mortality events in BLCA, KIRC, MESO, and UCS, but promoted them in LAML, LGG, and LUAD (Fig. S1A). Kaplan-Meier curves suggested better prognosis for high CD-NTase gene set scores in BLCA, KIRC, READ, and UCS, and worse in LAML, LUAD, and LGG (Fig. S1B-H). The diagnostic efficacy of the CD-NTase gene set was also examined across multiple tumors (Fig. S2). Notably, ESCA showed strong diagnostic efficacy with an AUC of 0.828, and AUCs exceeded 0.7 in other eight cancers.

Furthermore, to explore which immune cell types in pan-cancer might be influenced by the CD-NTase gene set, we calculated the immune cell infiltration levels in all TCGA samples and performed Spearman correlation analysis. As illustrated, the CD-NTase gene set was positively correlated with M1 macrophages, activated Dendritic cells, and Tregs, and negatively with memory B cells, Plasma cells, and CD4 naive T cells (Fig. S3A). Notably, M1 macrophages, which displayed strong microbicidal and tumoricidal activities and preferentially promoted inflammatory responses, showed the most significant correlation. Additionally, the CD-NTase gene set was significantly positively correlated with the immuneScore in most tumors (Fig. S3B). These findings suggest that the expression of the CD-NTase family in tumor cells might be involved in regulating the migration and infiltration of immune cells, thereby affecting the prognosis and immune therapy outcomes in human cancers. Considering the importance of PD-L1, TMB, and MSI as key biomarkers in immune therapy, correlations between TMB/MSI and CD-NTase gene set expression were also assessed across various cancer types. The results indicated a positive correlation between CD-NTase gene set expression and high TMB scores in BRCA, COAD, and STAD (Fig. S3C). Similarly, a positive correlation was observed between high MSI scores and CD-NTase gene set expression in COAD, LGG, and STAD (Fig. S3D). These results suggest that the CD-NTase family may serve as predictive biomarkers for the efficacy of cancer immune therapies in corresponding cancers.

### Genome-wide expression profiling of CD-NTase enzymes led to identify MB21D2 as a significant prognostic predictor in ESCC

**Figure 2:**
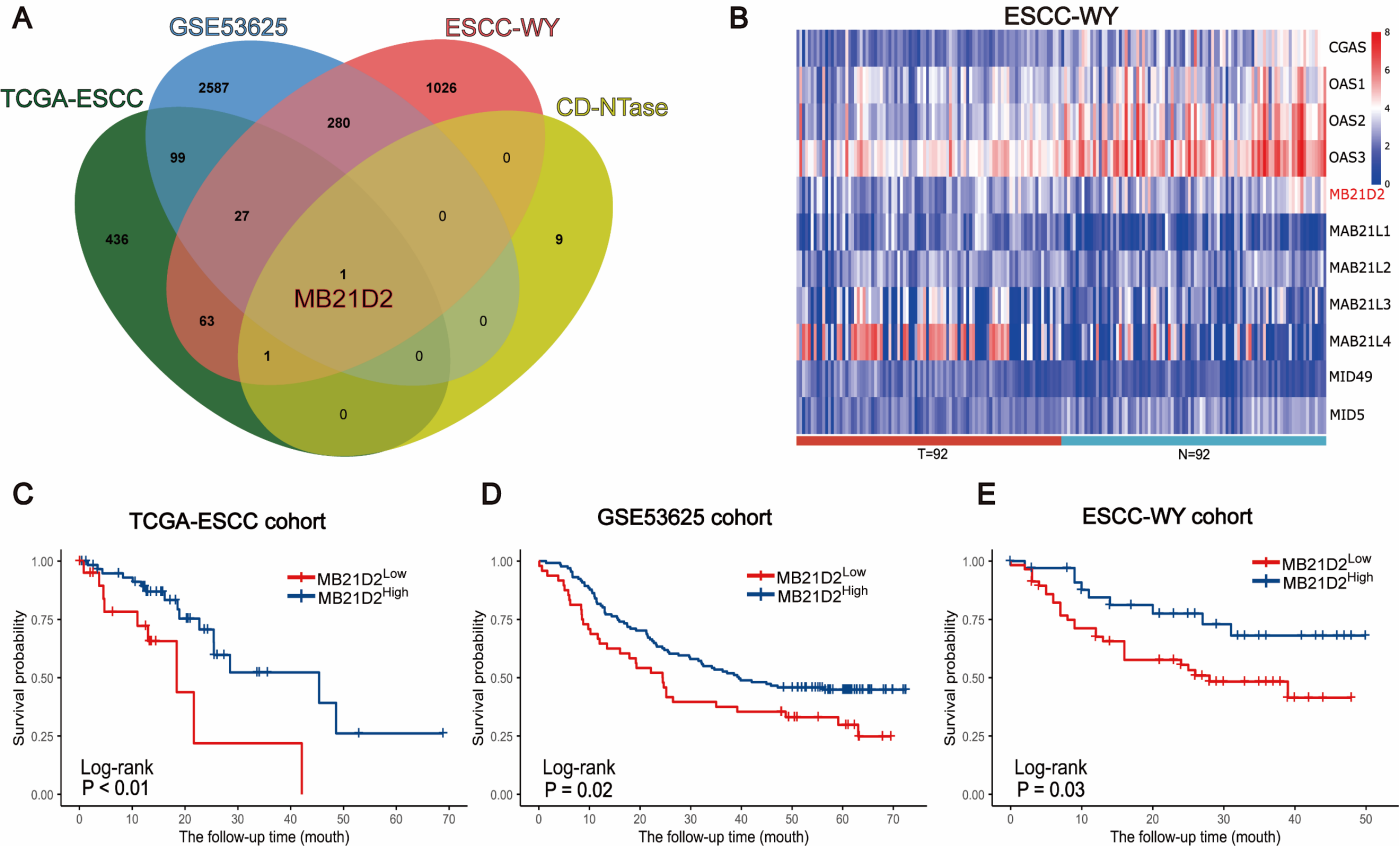
MB21D2 is significantly associated with prognosis in three ESCC clinical cohorts. **A** Venn diagram identified MB21D2 as the unique intersecting member among the 11 CD-NTase enzymes, signifying its importance as a prognostic marker in the TCGA-ESCC, GSE53625, and ESCC-WY clinical cohorts. **B** Heatmap analysis of mRNA related to the CD-NTase family members in the ESCC-WY cohort revealed distinct expression patterns in tumor patient tissue (T) and adjacent patient normal tissue (N). **C-E** OS analysis based on low MB21D2 expression (dichotomized by the best cutoff value for a significant difference) demonstrated its impact on ESCC patients in all three clinical cohorts by log-rank test: (**C**) TCGA (*P* < 0.01), (**D**) GSE53625 (*P* = 0.02), (**E**) ESCC-WY (*P* = 0.03).

Subsequently, we validated the clinical significance of CD-NTase enzymes in ESCC and explore their underlying mechanisms. To assess the clinical significance of CD-NTase enzymes in ESCC, we initially analyzed their expression using clinical public datasets as our training cohort. Data from 260 patients were collected, including 81 from TCGA and 179 ESCC samples from GSE53625. The GSE53625 dataset comprises 179 ESCC microarray samples from GSE53624 and GSE53622, containing 119 and 60 paired ESCC and adjacent normal tissue microarray expression profiles, respectively. The two datasets were combined and batch effects were removed as shown in Supplementary Fig. S4. Survival analysis integrating both public datasets revealed that MB21D2, a CD-NTase family member, was the only significant prognostic factor in both datasets (TCGA: P < 0.01, GSE53625: P = 0.02, Log-rank test; Fig. 2A-D). Based on this, we further evaluated the expression of MB21D2.

In the testing phase, MB21D2's clinical significance was assessed through RNA-seq in a clinical training cohort of 92 ESCC patients at our center. High and low expression groups in the validation cohort were determined using the same statistical model and cutoff threshold method as in the training cohort. Interestingly, patients with low MB21D2 expression in their tumors exhibited significantly worse OS compared to those with high MB21D2 levels (P=0.03, Log-rank test; Fig. 2A and e; Table S7). The mortality rates during the follow-up period were 37.1% for high MB21D2 expression patients and 47.4% for low MB21D2 expression patients.

### Successful validation of MB21D2 expression in clinical cohort highlights its prognostic importance

Based on survival analysis, patients were stratified into two groups using a cutoff value for MB21D2 expression. The relationships between MB21D2 expression and clinicopathological characteristics are delineated in Table 1. Subsequently, univariate and multivariate Cox proportional hazards analyses were conducted in two clinical training cohorts. Univariate analysis in the TCGA cohort indicated that low MB21D2 expression significantly correlates with OS (Hazard Ratio [HR] = 3.22, P < 0.01, Table 2), along with gender and tumor lymph node status. Moreover, multivariate analysis identified MB21D2 as a significant and independent prognostic factor for OS (HR = 2.5, P = 0.04, Table 2). Similarly, in the GSE53625 cohort, low MB21D2 expression was a notable prognostic factor for OS (HR = 1.61, P = 0.02, Table 2), with tumor status, lymph node status, and TNM staging also being significant. Again, multivariate analysis confirmed MB21D2's role as a crucial and independent predictor of OS (HR = 1.33, P = 0.02, Table 2). These findings substantiate the significant prognostic impact of MB21D2 in patient outcomes. Further analyses using clinical validation cohort data revealed that tumor status, positive lymph node status, TNM stage, and low MB21D2 expression were significantly associated with OS (P = 0.01, P < 0.01, P = 0.03, P = 0.03, respectively, Table 2). Additionally, multivariate analysis reiterated the importance of low tumor MB21D2 as an independent prognostic factor for OS (HR = 2.5, P = 0.02, Table 2), highlighting its clinical significance as a prognostic biomarker for ESCC patients.

Furthermore, we analyzed the prognostic value of MB21D2 across various cancers. Initially, we examined the association between MB21D2 expression levels and OS. Univariate Cox analysis of 33 cancers revealed that MB21D2 expression levels significantly impacted the prognosis in ACC, KIRC, LGG, LUAD, PCPG, and UCEC (Fig. S5A). Moreover, Kaplan-Meier curves indicated that high MB21D2 expression was associated with worse prognosis in UCEC and HNSC, but with better outcomes in LGG, KIRP, and KIRC (Fig. S5B-F). Additionally, MB21D2 expression was found to correlate with Progression-free interval (PFI). Forest plots demonstrated this association in ACC, CHOL, KIRC, LGG, LIHC, LUAD, and UCEC (Fig. S6A). Kaplan-Meier curves showed that high MB21D2 expression in CHOL, KIRC, and LGG was linked to a more favorable prognosis, whereas it correlated with a poorer prognosis in UCEC (Fig. S6B-E).

Finally, the relationship between MB21D2 expression levels and Disease-specific survival (DSS) was examined. Forest plots indicated a significant association of MB21D2 expression with DSS in ACC, KIRC, LGG, LUAD, and UCEC (Fig. S7A). Kaplan-Meier curves revealed that high MB21D2 expression led to better prognosis in KIRC, KIRP, and LGG, and to a worse prognosis in UCEC (Fig. S7B-E).

### Knockdown MB21D2 expression promotes proliferation, migration, and invasion in human ESCC cells

**Figure 3:**
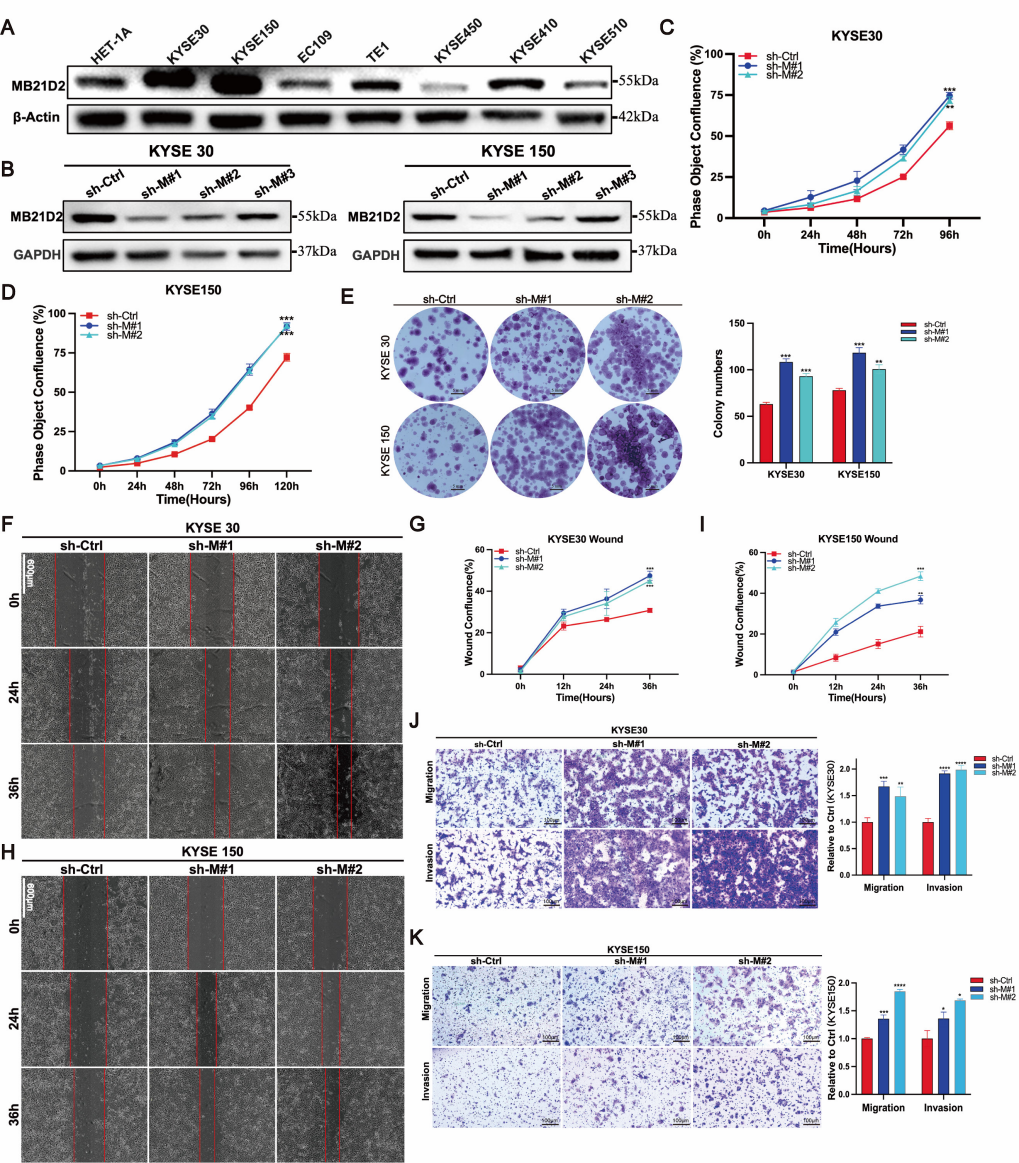
Knockdown of MB21D2 expression enhances proliferation, migration, and invasion in human ESCC cells *in vitro*. **A** The relative levels of proteins to β-Actin in human ESCC cell lines and non-tumorous esophageal epithelial cells were quantitatively analyzed via western blot. **B** The knockdown levels of MB21D2 in KYSE30 and KYSE150 were confirmed through western blot. **C-D** Enhanced proliferative abilities in KYSE30 (**C**) and KYSE150 (**D**) following MB21D2 knockdown were detected using Incucyte S3. **E** Knockdown of MB21D2 promoted clonogenic abilities in KYSE30 and KYSE150. **F-K** Cell migration in MB21D2 knockdown cell lines KYSE30 (**F-G, J**) and KYSE150 (**H-I, K**) was evaluated using scratch wound and *in vitro* transwell invasion assays. Data are presented as means ± S.D, reflecting three independent experiments. *P < 0.05, **P < 0.01, ***P < 0.001, ****P < 0.0001.

To elucidate the potential function of MB21D2 in ESCC cells, we initially examined its expression levels in the normal human esophageal epithelial cell line HET-1A and seven ESCC cell lines using western blot (Fig. 3A). Based on their relative expression levels, we selected KYSE30 and KYSE150 for the establishment of stable MB21D2 knockdown cell lines via lentiviral infection, confirmed by western blot (Fig. 3B) and fluorescence microscopy (Fig. S8A-B). Subsequently, we assessed cell proliferation using the Incucyte live-cell analysis system and clonogenic assays. Our results demonstrated that knockdown of MB21D2 significantly enhanced cell proliferation (Fig. 3C-D). *In vitro* colony formation assays revealed that knockdown of MB21D2 promoted clonogenic ability in ESCC cells (Fig. 3E). To further investigate the impact of MB21D2 knockdown on ESCC cell migration and invasion *in vitro*, we conducted wound healing and transwell assays. The results indicated a marked increase in wound closure speed, migration, and invasion capabilities in MB21D2-knockdown ESCC cells KYSE30 and KYSE150 compared to control cells (Fig. 3F-K). In summary, these experiments confirm the successful establishment of ESCC cell lines with stable overexpression and knockdown of MB21D2, demonstrating that silencing MB21D2 expression can enhance *in vitro* proliferation, migration, and invasion.

### Overexpression of MB21D2 inhibits ESCC cells proliferation, migration and wound-healing potential in human ESCC cells

**Figure 4:**
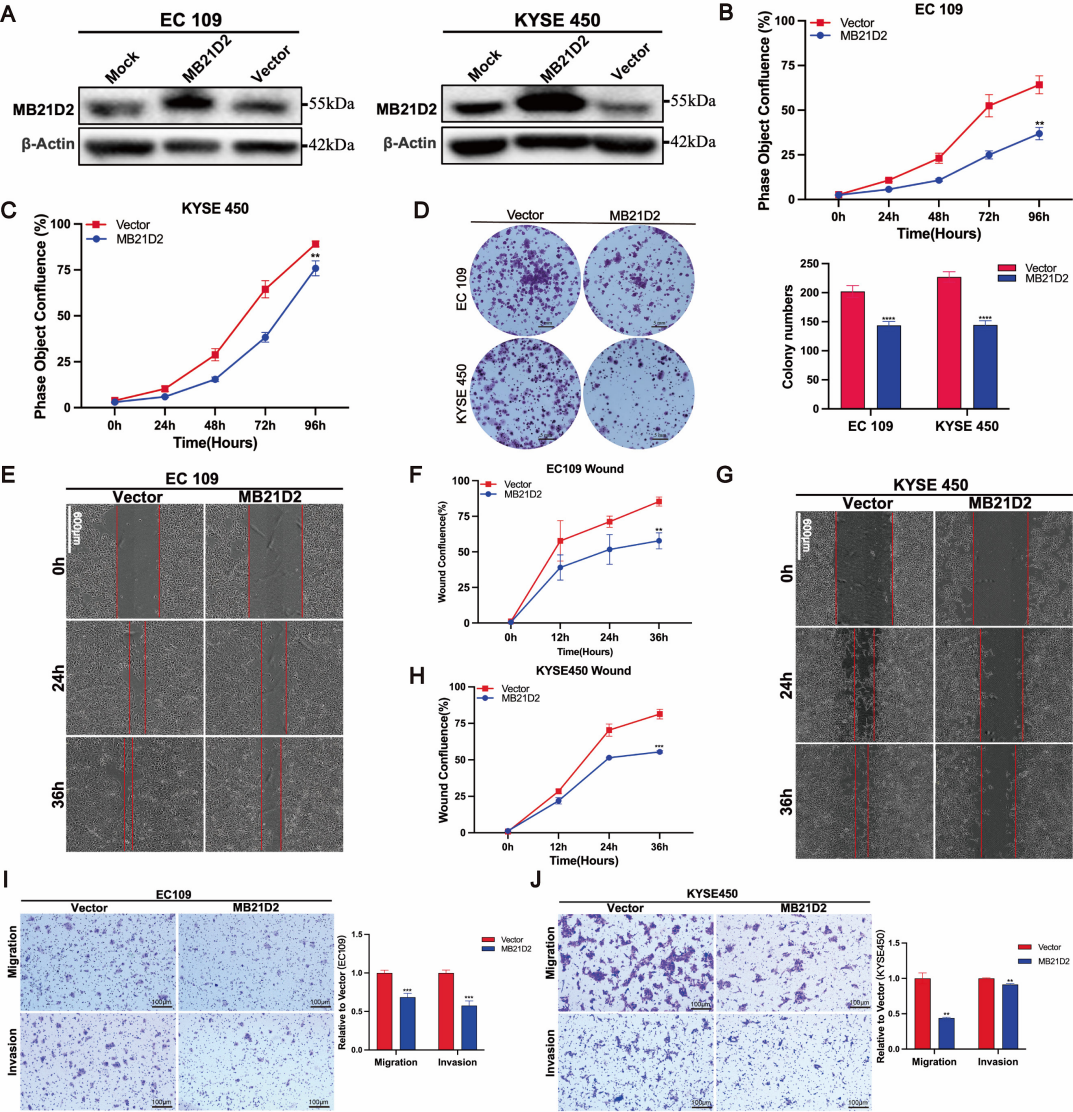
Overexpression of MB21D2 *in vitro* suppresses proliferation, migration, and invasion of ESCC cell lines. **A** MB21D2 overexpression levels in EC109 and KYSE450 were confirmed via western blot. **B-C** The Incucyte S3 system revealed diminished proliferative capabilities in EC109 (**B**) and KYSE450 (**C**) following MB21D2 overexpression compared to control groups.** D** Overexpression of MB21D2 reduced the clonogenic potential of both EC109 and KYSE450. **E-J** The migratory abilities of the MB21D2-overexpressing cell lines EC109 (**E-F, I**) and KYSE450 (**G-H, J**) were assessed and found to be impaired using wound healing and transwell invasion assays. Data are presented as means ± S.D, reflecting three independent experiments. **P < 0.01, ***P < 0.001, ****P < 0.0001.

Next, stable overexpression of MB21D2 in ESCC cell lines KYSE450 and EC109 was established through lentiviral transduction (Fig. S8C-D). The expression of MB21D2 protein was confirmed by western blot (Fig. 4A). Subsequently, cell functional assays were conducted on ESCC cells with stable overexpression of MB21D2. Compared to the control group, both types of MB21D2 overexpressing ESCC cells exhibited a significant reduction in proliferation rate (Fig. 4B and C). Additionally, clonogenic assays demonstrated a notable decrease in colony numbers in MB21D2 overexpressing cells compared to control (Fig. 4D). These data suggest that MB21D2 reduces the proliferation of ESCC cells. To assess the effects of MB21D2 on ESCC cell migration and invasion, scratch assays and transwell experiments were performed. In scratch assays, the migration rates of KYSE450 and EC109 cells with stable MB21D2 overexpression were significantly lower than those of the control group at 24 and 36 hours, indicating reduced cell migration (Fig. 4E-F and G-H). In transwell experiments, the number of cells penetrating the chamber membrane was substantially lower in MB21D2 overexpressing ESCC cells than in the control group, suggesting that MB21D2 diminishes ESCC cell migration and invasion abilities (Fig. 4I and J). Collectively, these results indicate that overexpression of MB21D2 suppresses the proliferation, migration, and invasion of ESCC cells, thereby potentially serving as a mechanism of action against ESCC *in vitro*.

### Stable knockdown of MB21D2 promotes growth and metastasis of ESCC cells *in vivo*

**Figure 5:**
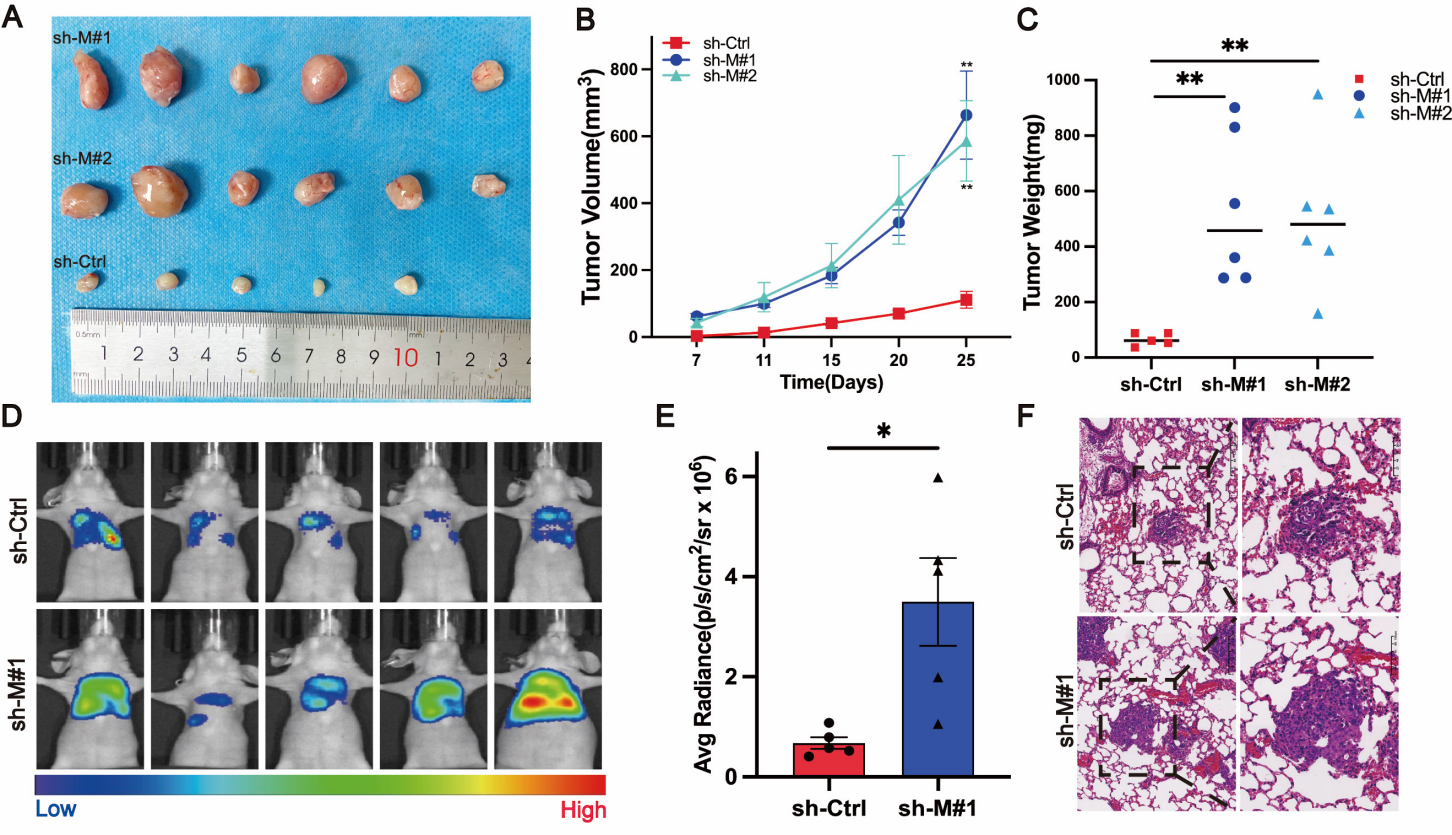
Stable knockdown of MB21D2 in KYSE30 cells promoted proliferation and distant metastasis *in vivo*. **A-C** BALB/c nude mice were inoculated with MB21D2-overexpressing KYSE30 cell lines to establish tumor xenograft models(**A**). After 25 days, the mice were euthanized, and the tumors were excised to measure volume changes (**B**) and weight (**C**), with statistical analysis performed (n=5 in the sh-Ctrl group, n=6 in the sh-M#1 and sh-M#2 group). **D-F** Additionally, 2x10^6^ luciferase-expressing sh-MB21D2 KYSE30 cells were intravenously injected into the nude mice (n=6 in sh-Ctrl and sh-M#1 group). At 8 weeks post-injection, the mice were anesthetized, and *in vivo* imaging was conducted using IVIS to assess fluorescence intensity (**D**), followed by statistical analysis (**E**). Paraffin-embedded sections of the lung (**F**) tissues from the nude mice were prepared, and histological hematoxylin and eosin (H&E) staining was employed to evaluate the presence of metastasis. Data are presented as means ± S.E.M. *P < 0.05, **P < 0.01.

In preliminary experiments, we established the *in vitro* effects of MB21D2 on ESCC cell biological functions. To further investigate MB21D2's *in vivo* impact on ESCC cells, we utilized immunodeficient mice (BALB/c nude mice) to develop xenograft and metastasis models. The mice, acquired and quarantined for a week, were subsequently housed in an SPF environment. In the xenograft model, KYSE30 cells, stably transfected with sh-MB21D2 (sh-M#1 and sh-M#2), or a scramble vector, were injected subcutaneously into the right axilla. Observations and measurements were conducted, culminating in euthanasia at 25 days post-injection for tumor size statistical analysis (Fig. 5A). Knockdown of MB21D2 significantly enhanced the growth of inoculated tumor cells *in vivo*, as evidenced by increased subcutaneous tumor volume (Fig. 5B) and weight (Fig. 5C) in the sh-MB21D2h groups compared to control. Furthermore, a nude mouse tail vein metastasis model revealed that MB21D2 depletion significantly augmented tumor cell metastasis to the lungs (Fig. 5D-F). These *in vivo* experiments suggest that silencing MB21D2 can promote ESCC cell growth and metastasis, implying that inhibiting MB21D2 expression may facilitate ESCC progression.

### Knockdown MB21D2 significantly activates the Wnt signaling pathway in ESCC cells

**Figure 6:**
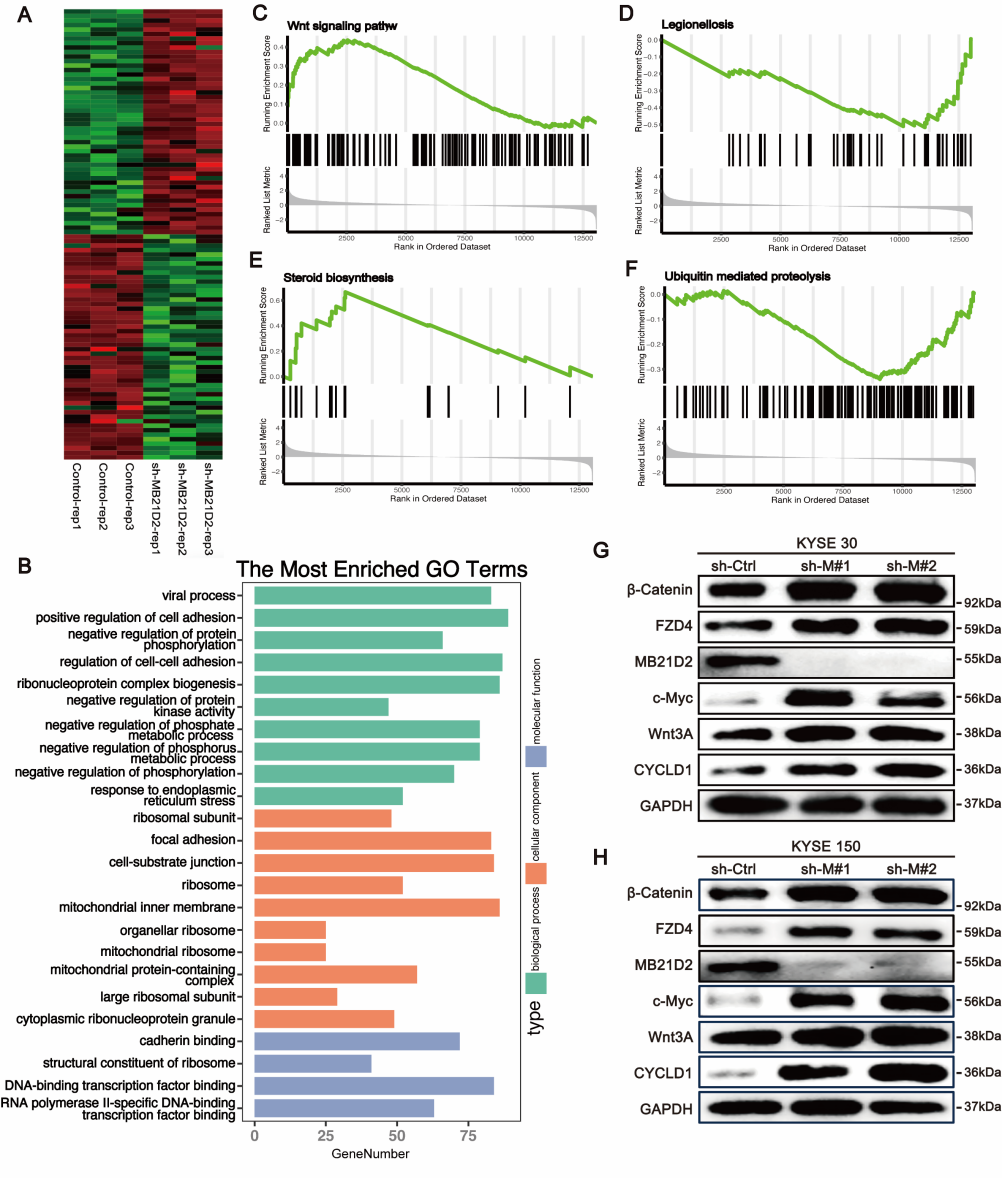
Knocking down MB21D2 in ESCC cells activates the Wnt/β-catenin signaling pathway, promoting tumor phenotypes. **A** Heatmap analysis was performed on the top 100 most significant DEGs between MB21D2 knockdown and control KYSE30 cells. **B** Gene Ontology (GO) analysis was applied to all differentially expressed genes.** C-F** Gene Set Enrichment Analysis (GSEA) revealed that MB21D2 activation or inhibition significantly activated the Wnt signaling(**C**) and steroid biosynthesis pathways(**D**), while notably inhibiting Legionella-related pathways(**E**) and ubiquitin-mediated protein degradation(**F**). **G-H** Western blot assays were used to examine proteins related to the Wnt pathway in MB21D2 knockdown KYSE30 and KYSE150.

To further comprehend the biological significance and potential mechanisms of MB21D2 in ESCC, we conducted high-throughput transcriptomic sequencing to investigate how MB21D2 knockdown alters mRNA transcription levels in KYSE30 cells (Fig. 6A). DEGs were identified using a P-value cutoff of < 0.05, revealing 2439 DEGs, with 1318 upregulated and 1121 downregulated (Table S8). GO analysis indicated that these DEGs primarily participate in biological functions such as cell adhesion, ribosomal protein complex formation, viral processes, negative regulation of phosphorus metabolism, and protein phosphorylation (Fig. 6B). Upregulated genes were mainly involved in cell adhesion, protein synthesis, and inflammatory responses to injury (Fig. S9A), whereas downregulated genes were associated with cellular catabolic processes, protein ubiquitination, and viral processes (Fig. S9B). KEGG analysis revealed that these DEGs were predominantly enriched in pathways including the cell cycle, endocytosis, cell adhesion, ferroptosis, the Hippo pathway, and the TGF-β pathway (Fig. S9C). Additionally, GSEA analysis indicated significant activation of the steroid biosynthesis and Wnt signaling pathways, with notable suppression of Legionella-related pathways and ubiquitin-mediated proteolysis (Fig. 6C-F). These results suggest a close association between MB21D2 and cell adhesion, as well as the Wnt signaling pathway in ESCC cells. To further validate the regulatory effects of MB21D2 on genes related to the Wnt signaling pathway, we examined Wnt pathway-related proteins in MB21D2 knockdown KYSE 30 and KYSE150 cells, revealing that MB21D2 silencing activates the expression of proteins associated with the Wnt signaling pathway (Fig. 6G).

### Inhibitors of the Wnt/β-catenin signaling pathway can reverse the effects of MB21D2 silencing on the proliferation, migration, and invasion of ESCC cells

**Figure 7:**
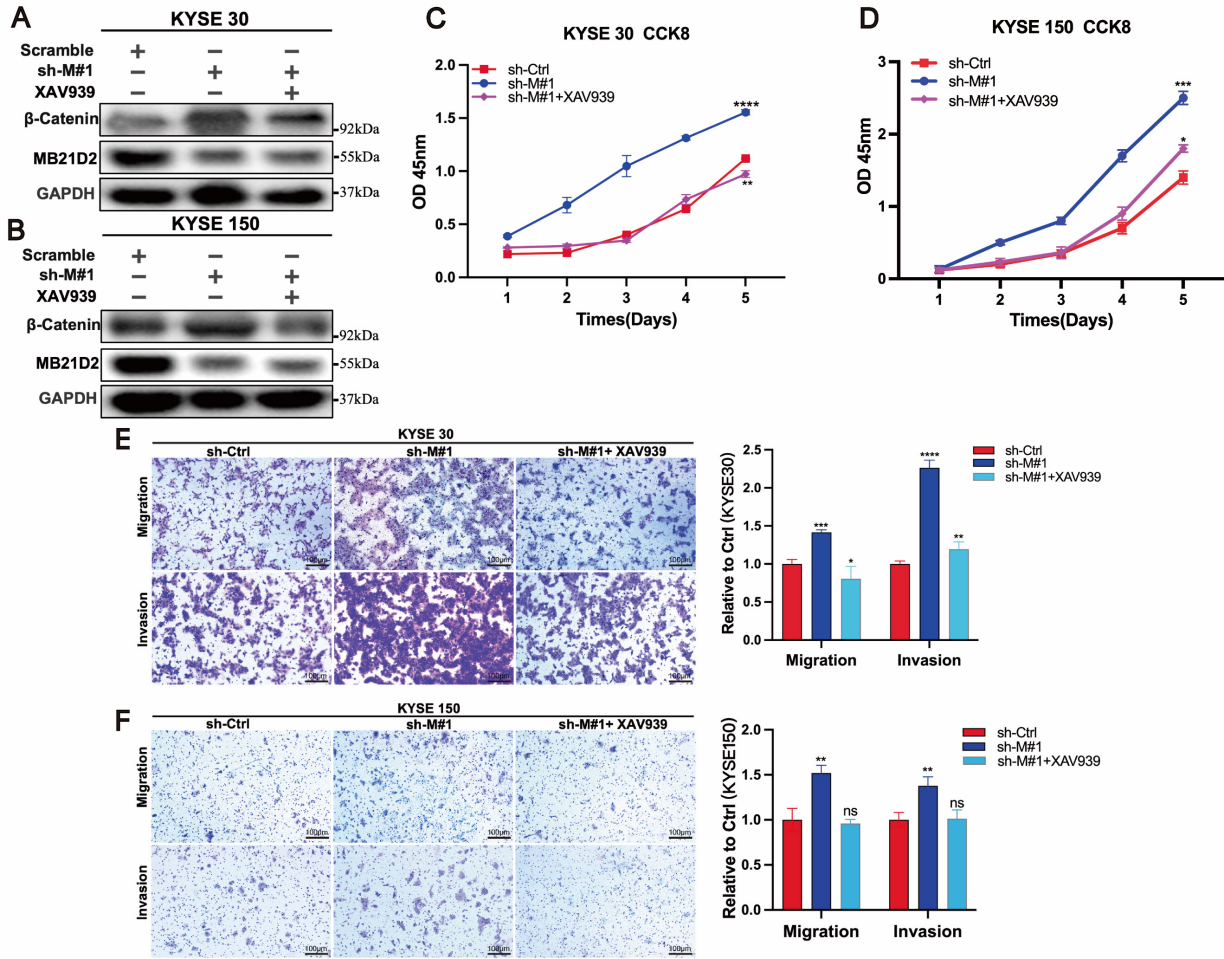
Inhibition of the Wnt/β-catenin signaling pathway reverses the effects of MB21D2 knockdown on the proliferation, migration, and invasion of ESCC cells. **A-B** β-catenin protein levels altered by MB21D2 knockdown were restored by XAV939 in KYSE30(**A**) and KYSE150 cells(**B**). **C-D** Inhibiting the Wnt/β-catenin pathway counteracted the impact of MB21D2 knockdown on the proliferative activity of KYSE30 (**C**) and KYSE150 (**D**) cells by CCK8. **E-F** Inhibitors of the Wnt/β-catenin signaling pathway mitigated the effects of MB21D2 knockdown on the migration and invasion of KYSE30 (**E**) and KYSE150 (**F**) cells by vitro transwell invasion assays. Data are presented as means ± S.D, reflecting three independent experiments. *P < 0.05, **P < 0.01, ***P < 0.001, ****P < 0.0001, ns: nonsignificant.

Previous studies have demonstrated that silencing MB21D2 increases the expression of β-catenin, a key molecule in the Wnt/β-catenin signaling pathway, and promotes proliferation, migration, and invasion in KYSE30 and KYSE150 cells. To further validate the role of MB21D2 in regulating the biological functions of ESCC cells through the Wnt/β-catenin pathway, we conducted rescue experiments using the Wnt/β-catenin pathway inhibitor XAV939. Western blot analysis confirmed that XAV939 reversed the effects of MB21D2 silencing on β-catenin protein levels in KYSE30 and KYSE150 cells (Fig. 7A and B). Additionally, XAV939 restored the proliferative activity in these cells affected by MB21D2 (Fig. 7C and D). Transwell assays further indicated that XAV939 could reverse the impact of MB21D2 on the migration and invasion abilities of KYSE30 and KYSE150 cells (Fig. 7E and F). Therefore, we conclude that MB21D2 regulates the proliferation, migration, and invasion of ESCC cells via the Wnt/β-catenin signaling pathway.

## Discussion

In this study, we conducted a comprehensive and systematic investigation to elucidate the role of CD-NTase family members in ESCC. Our research is the first to demonstrate the significant clinical importance of MB21D2 in ESCC. Previous studies have indicated that CD-NTases exhibits diverse physiological and pathological roles in humans, predominantly in activating immune responses 13,21-23. However, research on CD-NTases in tumor biology remains scarce. Through integrative bioinformatics, *in vitro*, and *in vivo* experiments, we demonstrated that low MB21D2 expression correlates with poor survival outcomes and increased tumor aggressiveness via activation of the Wnt/β-catenin signaling pathway. These findings provide valuable insights into the molecular mechanisms underlying ESCC progression and potential therapeutic targets.

MB21D2, a Mab21 domain-containing protein, is part of the CD-NTase enzymes. Beyond embryonic development 23,24, Mab21 homologs may influence pathways closely related to tumorigenesis and progression, such as DNA repair 25,26, apoptosis 27, and inflammatory responses 28. Recent discoveries using proximity biotinylation and quantitative proteomics suggest that Mab21 domain-containing proteins may act as novel components of the E-cadherin adhesome 29. MB21D2 has been implicated in immune regulation through the cGAS-STING pathway, where it enhances type I interferon production in response to cytosolic DNA sensing 29. However, its role in cancer remains underexplored. Our study reveals that MB21D2 suppresses ESCC progression by modulating the Wnt/β-catenin pathway, a central axis in cell proliferation, migration, and invasion contrasts 16 with prior findings suggesting a pro-oncogenic role for Q311E mutant MB21D2 in head and neck squamous cell carcinoma (HNSCC), where its overexpression promoted malignancy. This underscores potential cancer-type-specific functions, possibly due to different tumor microenvironments or interaction networks.

In ESCC cells with MB21D2 knockdown, we observed activation of the Wnt/β-catenin pathway. Aberrant activation of Wnt/β-catenin signaling is closely associated with increased cancer incidence, progression of malignancy, poor prognosis, and even higher cancer-related mortality rates. In the classical Wnt/β-catenin pathway, dysregulation of the transcription factor β-catenin is an early event in tumorigenesis 30. Wnt3A, a potent activator of Wnt/β-catenin signaling, is involved in the progression of many cancers. For instance, in solid tumors, Wnt3A has been shown to promote the development and progression of colorectal cancer (CRC) 31, prostate cancer 32, liver cancer 33, and lung cancer 34. In this study, our findings reveal that MB21D2 knockdown increases the expression of Wnt3A, β-catenin, and other related factors in the Wnt/β-catenin pathway, promoting a malignant phenotype in ESCC cells. Of course, the molecular mechanisms linking MB21D2 to Wnt/β-catenin pathway regulation require further exploration at the protein-interaction level. Furthermore, exploring MB21D2's role in modulating tumor immunity through the cGAS-STING axis in immune-competent models may reveal new therapeutic strategies combining immune checkpoint inhibitors with Wnt pathway inhibitors.

In summary, our study elucidates the clinical significance of MB21D2 expression in ESCC and demonstrates MB21D2 modulates the biological functions of ESCC by influencing the Wnt/β-catenin signaling pathway at the cellular level. This evidence suggests that overexpression of MB21D2 could represent a novel therapeutic mechanism to reduce the malignant phenotype of ESCC by inhibiting the Wnt/β-catenin signaling pathway, thus providing a new theoretical foundation and data support for future ESCC treatments.

## Conclusions

Our study provides compelling evidence that MB21D2 acts as a tumor suppressor in ESCC by negatively regulating the Wnt/β-catenin signaling pathway. These findings offer a promising avenue for developing targeted therapies aimed at restoring MB21D2 expression or inhibiting Wnt pathway activation, thereby improving outcomes for ESCC patients.

## Supplementary Material

Supplementary figures and tables.

## Figures and Tables

**Table 1:** Comparison of clinicopathological characteristics according to MB21D2 level

Characteristics	TCGA-ESCC cohort				GEO-ESCC cohort				WY-ESCC cohort			
	Total	MB21D2 level			Total	MB21D2 level			Total	MB21D2 level		
	n=81	Low	High	*P*-value^a^	n=179	Low	High	*P*-value^a^	n=92	Low	High	*P*-value^a^
		n=21	n=60			n=48	n=131			n=57	n=35	
Age				0.32				0.30				0.72
<60	48	10	38		99	23	76		35	23	12	
≥60	33	11	22		80	25	55		57	34	23	
Gender				0.66^b^				0.78				0.54
Male	69	19	50		146	38	108		69	41	28	
Female	12	2	10		33	10	23		23	16	7	
Tumor stage				0.67				0.10				0.72
T1+T2	36	8	28		39	15	24		27	18	9	
T3+T4	45	13	32		140	33	107		65	39	26	
Nodes metastasis				0.81^b^				0.27				0.58
yes	30	9	21		96	22	74		52	34	18	
no	43	10	33		83	26	57		40	23	17	
Not available	8	2	6									
Grade				0.57^b^				0.68				**< 0.01^b^**
well	15	3	12		98	26	72		3	2	1	
moderately	38	10	28		49	15	34		41	18	23	
poorly	19	7	12		32	7	25		45	35	10	
Not available	9	1	8						3	2	1	
TNM stage				**0.01**				0.08				0.75
I-II	51	8	43		87	29	58		31	18	13	
III-IV	30	13	17		92	19	73		61	39	22	
Tumor location				*NA*				0.52				0.77
Lower	-	-	-		62	14	48		47	28	19	
Middle	-	-	-		97	27	70		29	20	9	
Upper	-	-	-		20	7	13		16	9	7	
Vascular invasion				*NA*				*NA*				**0.04**
yes	-	-	-		-	-	-		32	25	7	
no	-	-	-		-	-	-		60	32	28	
Perineural invasion				*NA*				*NA*				0.29
yes	-	-	-		-	-	-		42	29	13	
no	-	-	-		-	-	-		50	28	22	
Drinking				1				0.09				1
yes	60	16	44		106	23	83		39	24	15	
no	21	5	16		73	25	48		53	33	20	
Smoking				0.56^c^				0.28				0.20
yes	78	21	57		114	27	87		43	31	12	
no	3	0	3		65	21	44		49	28	21	

Statistically significant *P*-values are bold. ^a^ Chi-square test, ^b^ Yates's correction for continuity, ^c^ Fisher's exact test. *NA* not available

**Table 2:** Univariate and multivariate analysis of OS using Cox-regression model

	Univariate analysis			Multivariate analysis		
Characteristics	HR	95% CI	*P*-value	HR	95% CI	*P*-value
**TCGA-ESCC cohort**						
Age (≥ 60 vs. < 60)	1.16	0.47-2.84	0.74			
Gender (Male vs. Female)	8.38	1.1-64.57	**0.04**	6.58	0.83-52.27	0.08
Tumor status (T3-4 vs. T1-2)	1.08	0.46-2.56	0.86			
Nodes metastasis (yes vs. no)	2.55	1.05-6.19	**0.04**	1.71	0.7-4.16	0.24
TNM stage (III+IV vs. I+II)	2.33	0.98-5.51	0.06			
MB21D2 status (low vs. high)	3.22	1.35-7.69	**< 0.01**	2.5	1.06-5.88	**0.04**
**GEO-ESCC cohort**						
Age (≥ 60 vs. < 60)	1.68	1.15-2.46	**< 0.01**	1.53	1.04-2.24	**0.03**
Gender (Male vs. Female)	0.78	0.50-1.25	0.307			
Tumor status (T3-4 vs. T1-2)	1.09	0.69-1.73	0.712			
Nodes metastasis (yes vs. no)	2.13	1.42-3.19	**< 0.01**	1.41	0.76-2.6	0.28
TNM stage (III+IV vs. I+II)	2.16	1.45-3.2	**< 0.01**	1.68	0.92-3.06	0.09
MB21D2 status (low vs. high)	1.61	1.08-2.44	**0.02**	1.33	1.08-2.44	**0.02**
**WY-ESCC cohort**						
Age (≥ 60 vs. < 60)	1.16	0.59-2.28	0.67			
Gender (Male vs. Female)	2.26	0.94-5.43	0.07			
Tumor status (T3-4 vs. T1-2)	3.34	1.30-8.57	**0.01**	3.14	1.13-8.77	**0.03**
Nodes metastasis (yes vs. no)	2.64	1.28-5.48	**< 0.01**	2.16	0.65-7.14	0.21
TNM stage (III+IV vs. I+II)	2.46	1.08-5.61	**0.03**	0.79	0.19-3.19	0.74
MB21D2 status (low vs. high)	2.27	1.08-4.76	**0.03**	2.5	1.18-5.26	**0.02**

Statistically significant *P*-values are bold. HR hazard ratio, CI confidence interval.
