# Supplementary material for: Clinical and Functional Characterization of CD-NTase Enzymes in Esophageal Squamous Cell Carcinoma
Source: J Cancer. 2025 Jun 12;16(9):2822–36. doi: 10.7150/jca.100226 (PMC12244066; doi:10.7150/jca.100226)
Supplement: Supplementary file 1 — Supplementary figures and tables. [file jcav16p2822s1.zip › Supplementary materials/Supplementary Figure.docx]

## Supplementary Figure


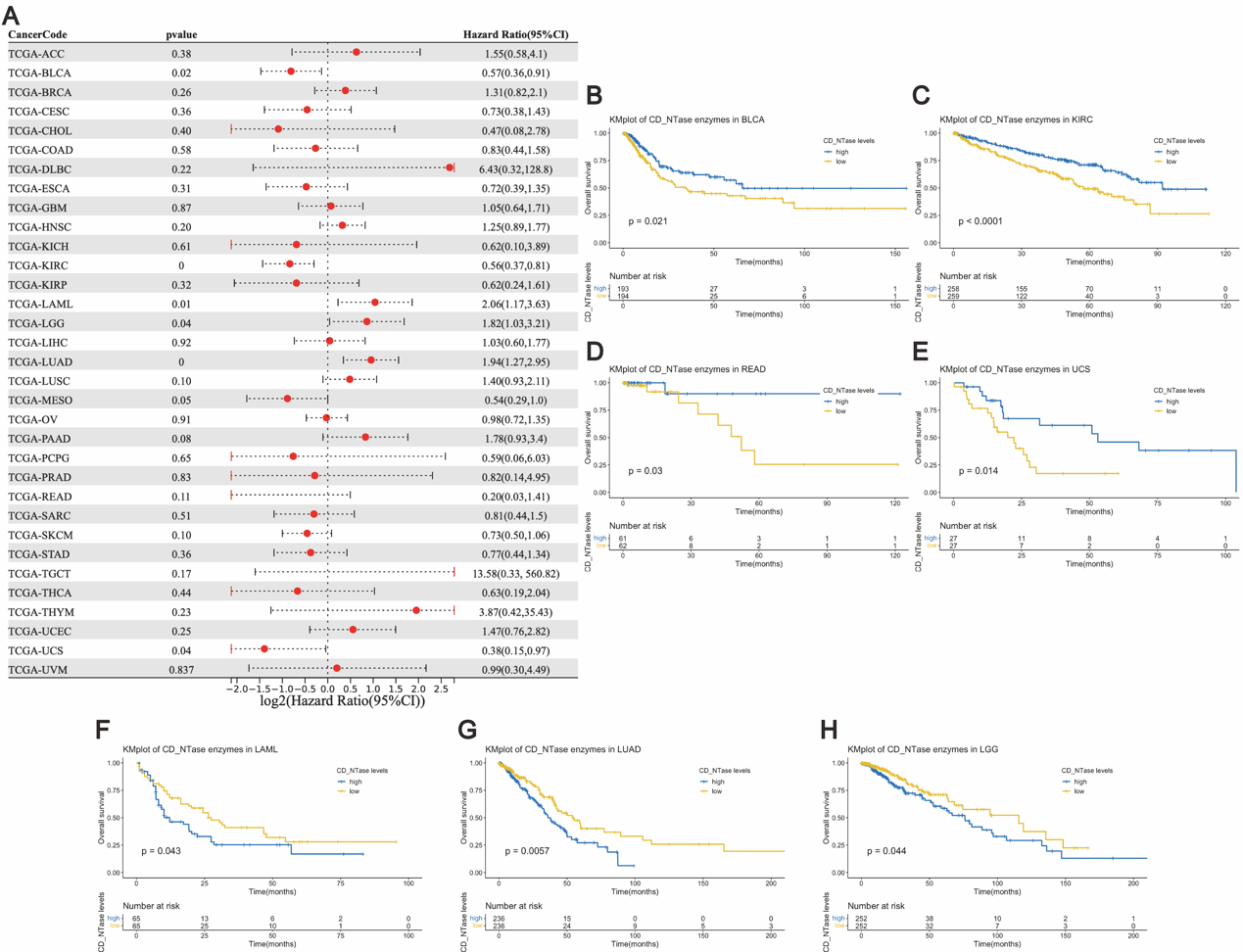


**Fig. S1** The association between the CD-NTase gene set and Overall Survival (OS) within the TCGA database was examined. (**A**) Univariate Cox proportional-hazards model, based on the TCGA database, was utilized to analyze the correlation between the CD-NTase gene set and 33 types of tumors. (**B-H**) The CD-NTase gene set score was categorized into high and low groups for Kaplan–Meier curve analysis. Significant differences in OS were observed in seven tumor types (*P* < 0.05).


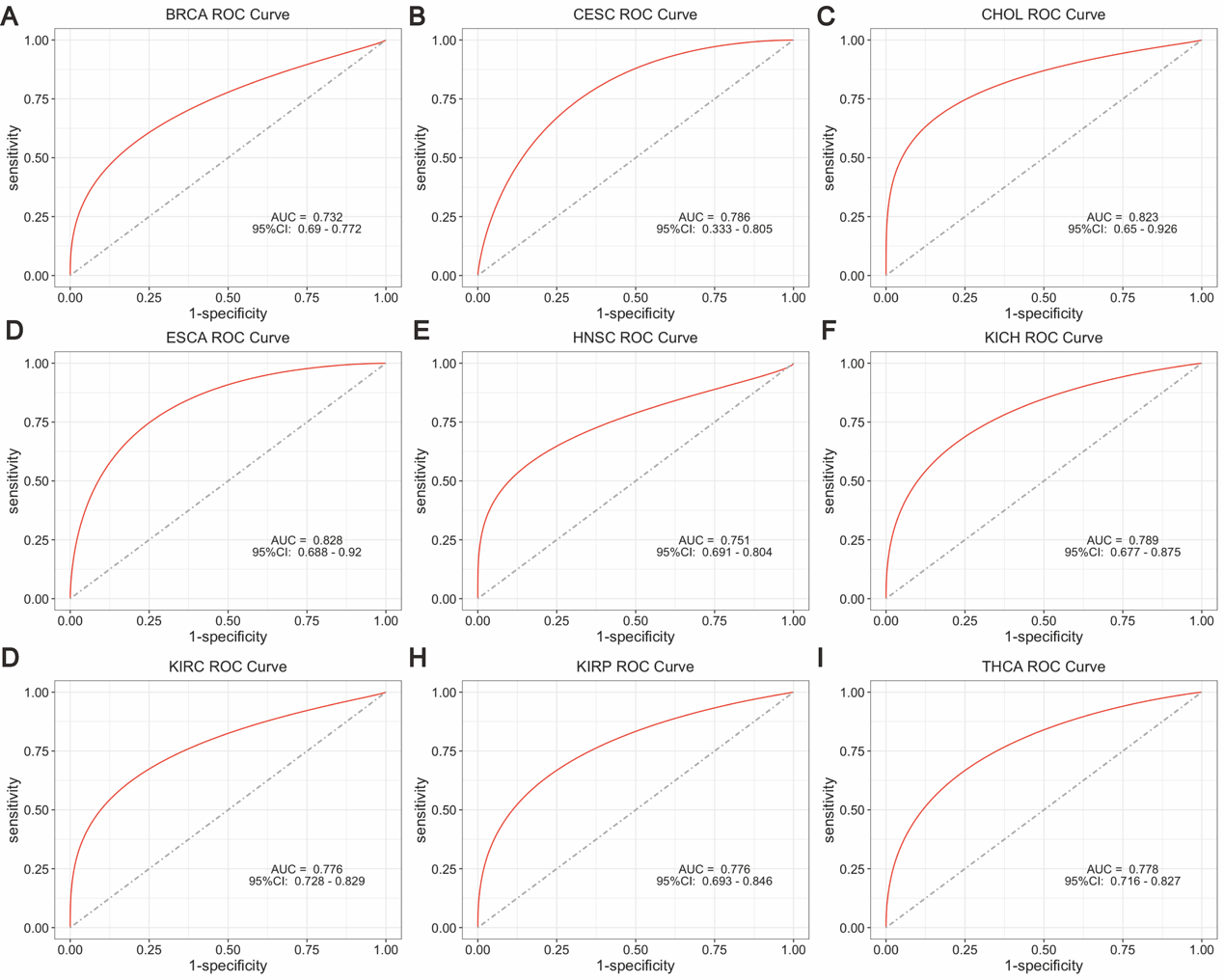


**Fig. S2** The Receiver Operating Characteristic (ROC) curve analysis of the CD-NTase gene set across 22 different cancer types demonstrates its diagnostic value. Notably, in nine cancer types – (**A**) BRCA, (**B**) CESC, (**C**) CHOL, (**D**) ESCA, (**E**) HNSC, (**F**) KICH, (**G**) KIRC, (**H**) KIRP, (**I**) THCA – the area under the ROC curve (AUC) exceeds 0.7, indicating the gene set's significant diagnostic efficacy (this analysis only includes results where the AUC is greater than 0.7).


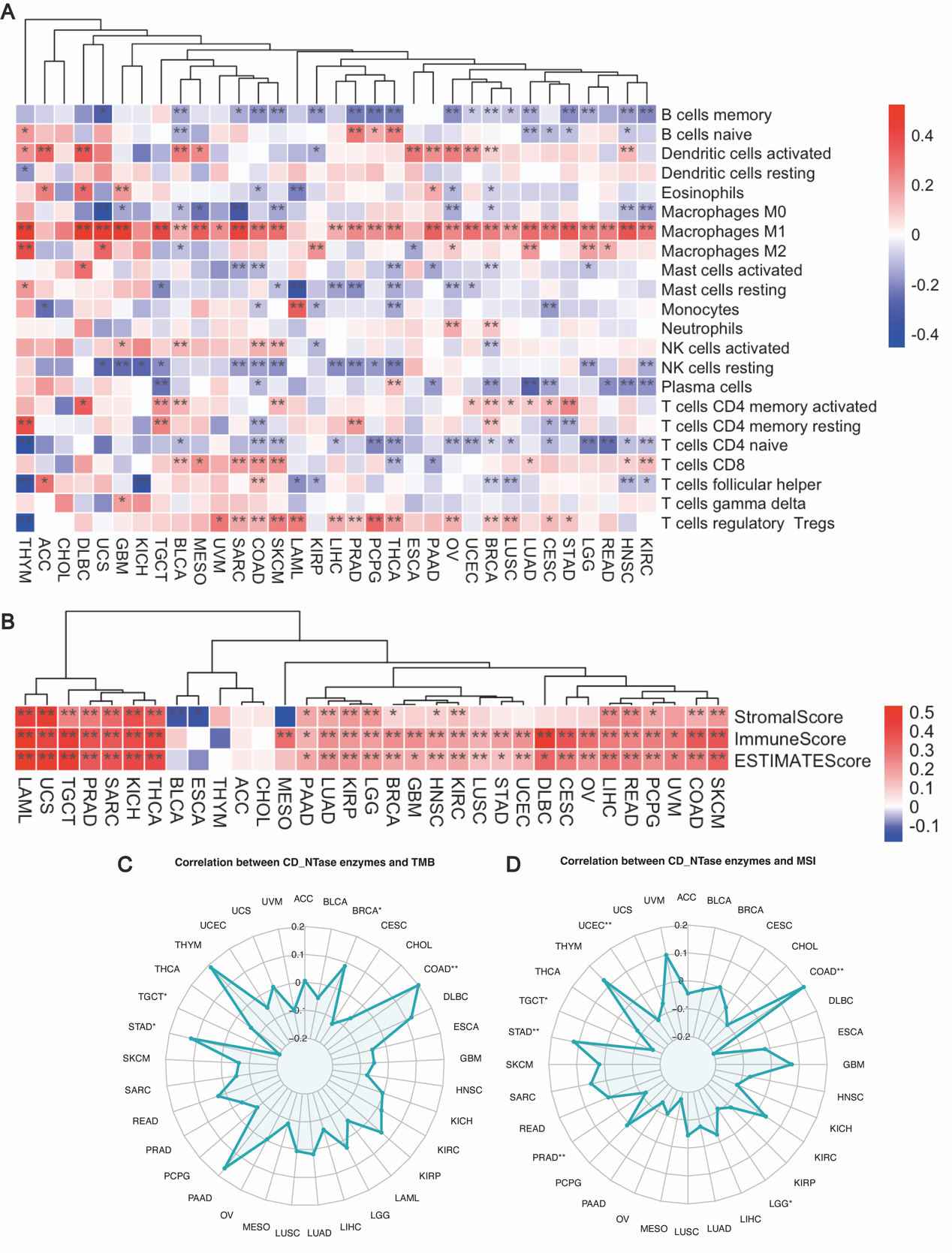


**Fig. S3** The study conducted a pan-cancer analysis to examine the correlations between the CD-NTase gene set and immune cell infiltration ratio, immune score, microsatellite instability (MSI), and tumor mutation burden (TMB). (**A**) Heatmap illustrating the correlation between the CD-NTase gene set and immune cell ratio. (**B**) Heatmap displaying the correlation between the CD-NTase gene set and the immune score. (**C**) Radar chart showing the correlation between the CD-NTase gene set and microsatellite instability (MSI). (**D**) Radar chart demonstrating the correlation between the CD-NTase gene set and tumor mutation burden (TMB). Red squares indicate a positive correlation and blue squares a negative correlation. Empty squares denote a lack of significant correlation. Statistical significance is indicated with **P* < 0.05 and ***P* < 0.01.


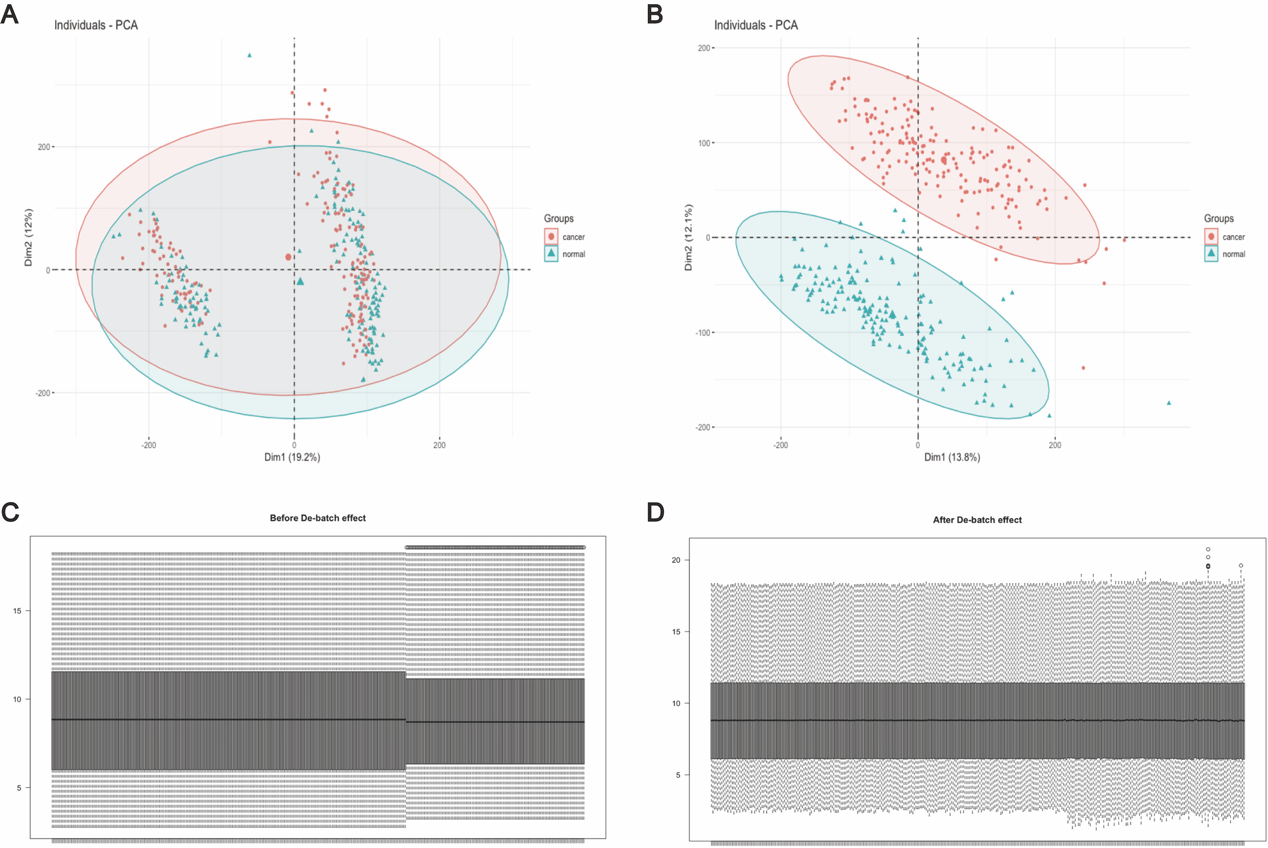


**Fig. S4** The microarray expression profiles from the GSE53624 and GSE53622 were merged, and batch effects were corrected using the “ComBat” function from the “sva” package in R. Figures illustrate the data before and after batch effect correction. (**A**) PCA plot before batch effect correction, (**B**) PCA plot after batch effect correction. (**C**) Box plot of expression levels before batch effect correction, and (**D**) Box plot of expression levels after batch effect correction.


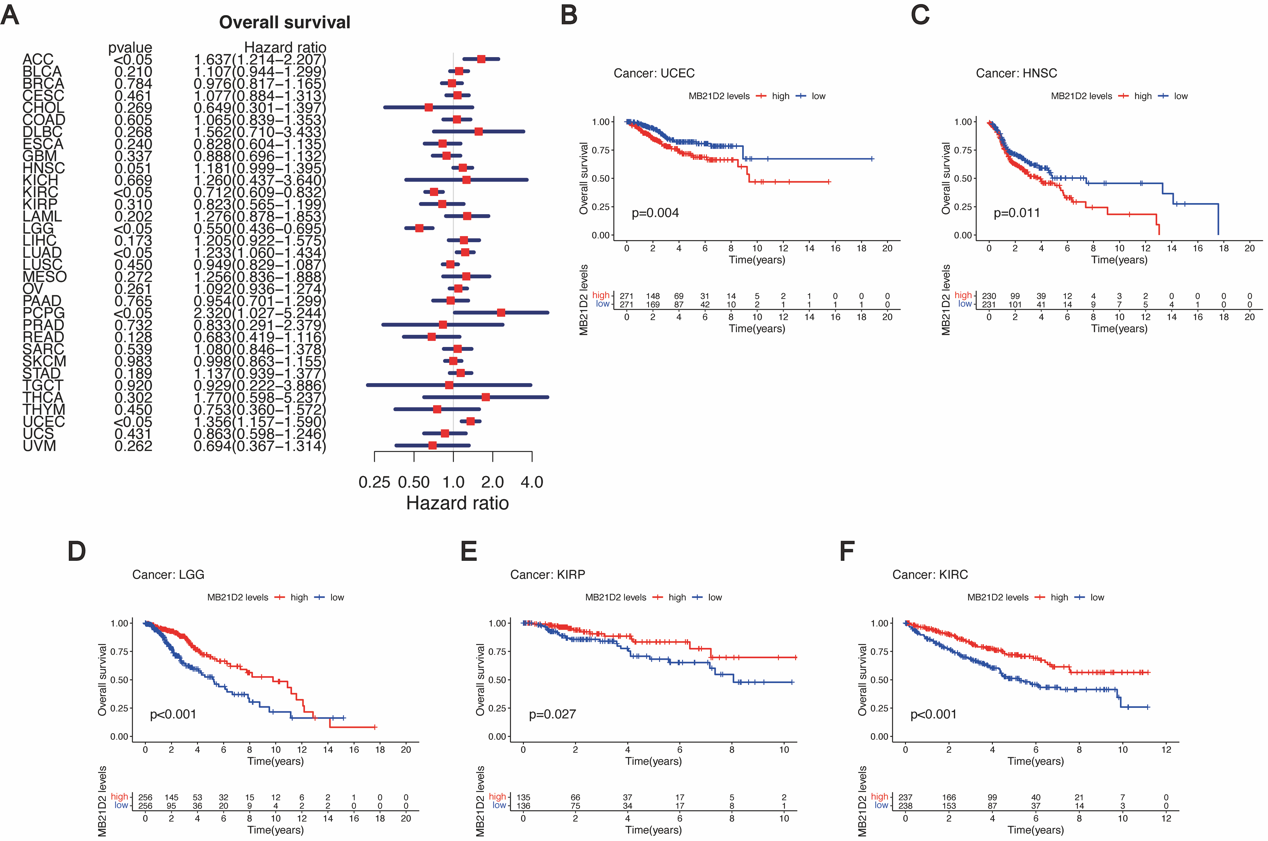


**Fig. S5** Association between MB21D2 expression and OS in TCGA Database. (**A**) Analysis of the relationship between MB21D2 expression and OS across various cancer types using univariate Cox regression based on TCGA Database. (**B-F**) Division of each cancer type in TCGA into high and low MB21D2 expression groups to explore their association with OS. Significant differences in OS were observed in five tumor types (*P* < 0.05).


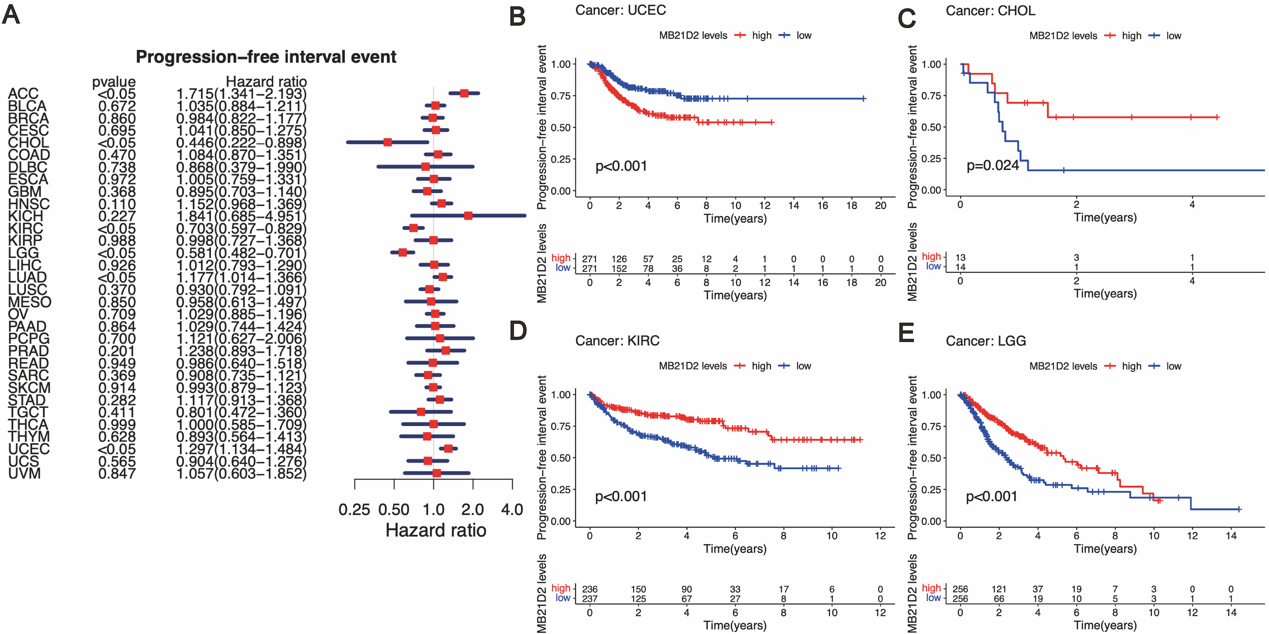


**Fig. S6** Correlation between MB21D2 expression and Progression Free Interval (PFI) in TCGA Database. (**A**) Analysis of the relationship between MB21D2 expression levels and PFI across various cancer types using univariate Cox regression based on TCGA Database. (**B-E**) Categorization of each cancer type in TCGA into high and low MB21D2 expression groups to examine their correlation with PFI. Significant differences in PFI were observed in four tumor types (*P* < 0.05).


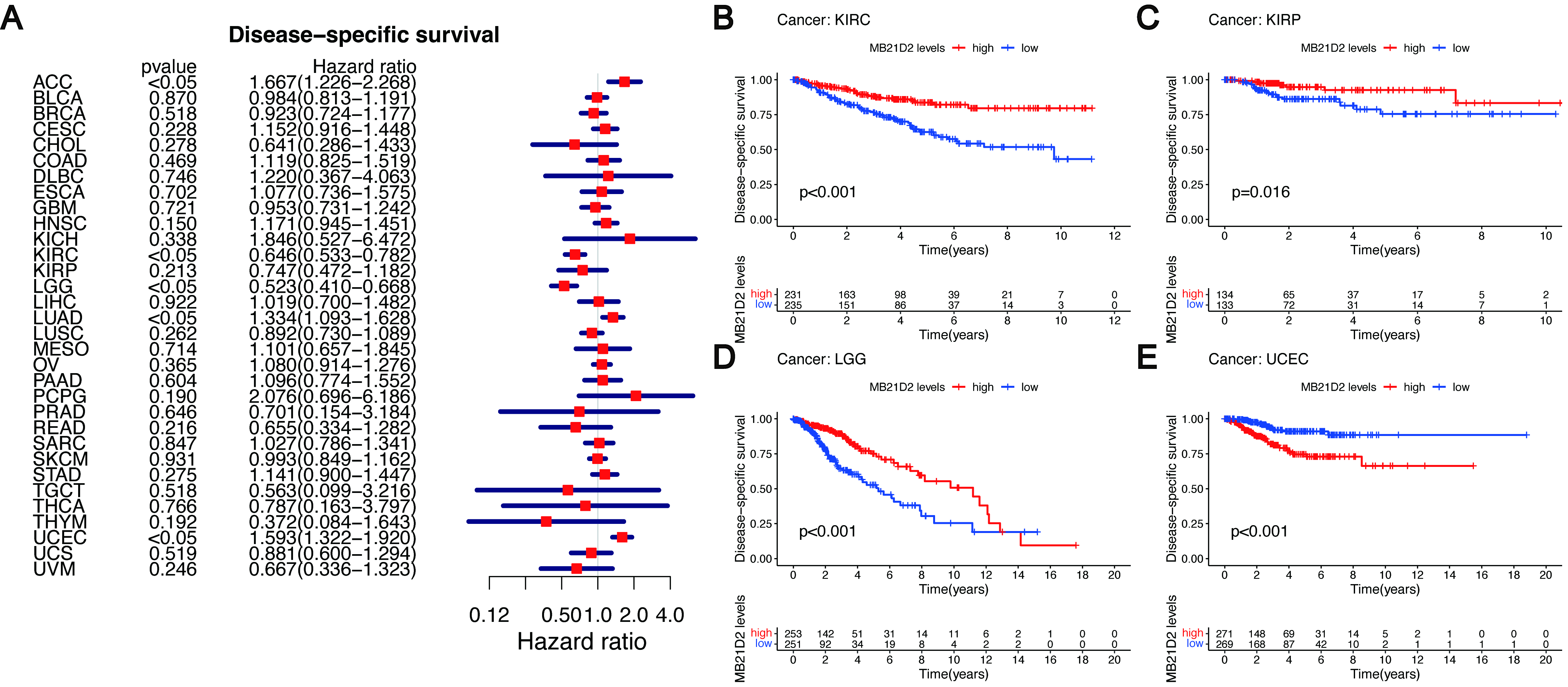


**Fig. S7** Relationship between MB21D2 Expression and Disease Specific Survival(DSS) in TCGA Database. (**A**) Investigation of the association between MB21D2 expression and DSS in various cancer types through univariate Cox regression using TCGA Database. (**B-E**) Division of each cancer type in TCGA into high and low MB21D2 expression groups for analysis of their relationship with DSS. Significant differences in DSS were observed in four tumor types (*P* < 0.05).


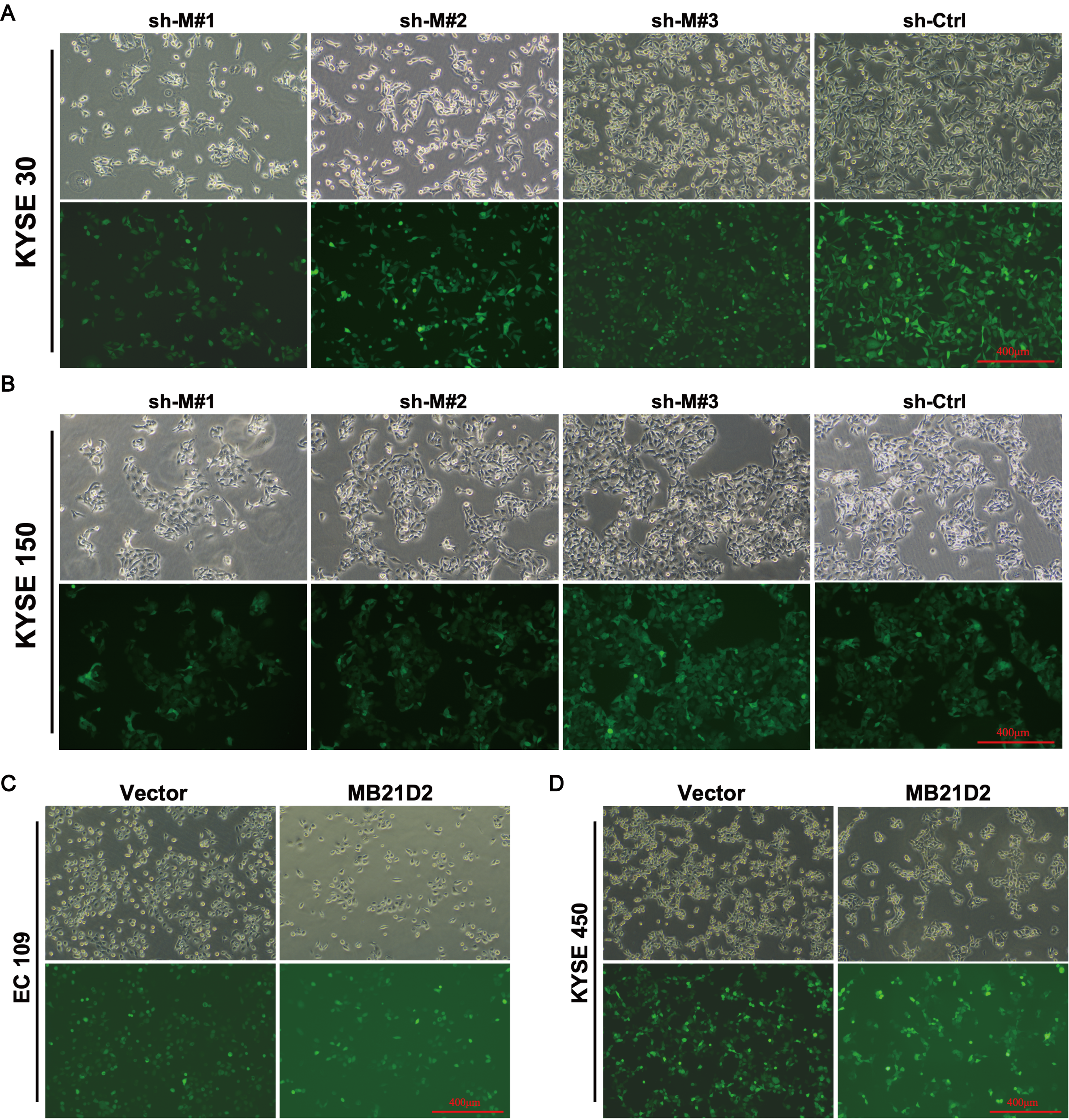


**Fig. S8** The plasmids were engineered to include a GFP fluorescent segment, enabling the assessment of lentiviral infection efficiency via fluorescence microscopy. (**A-B**) GFP fluorescence images post-lentiviral infection of KYSE30 and KYSE150 cells with knockdown viruses, including three sh-MB21D2 variants (sh-M#1, sh-M#2, and sh-M#3). The scrambled group (sh-Ctrl) served as a negative control. (**C-D**) GFP fluorescence images following overexpression lentiviral infection in EC109 and KYSE450 cells. Empty vectors were used as the control group.


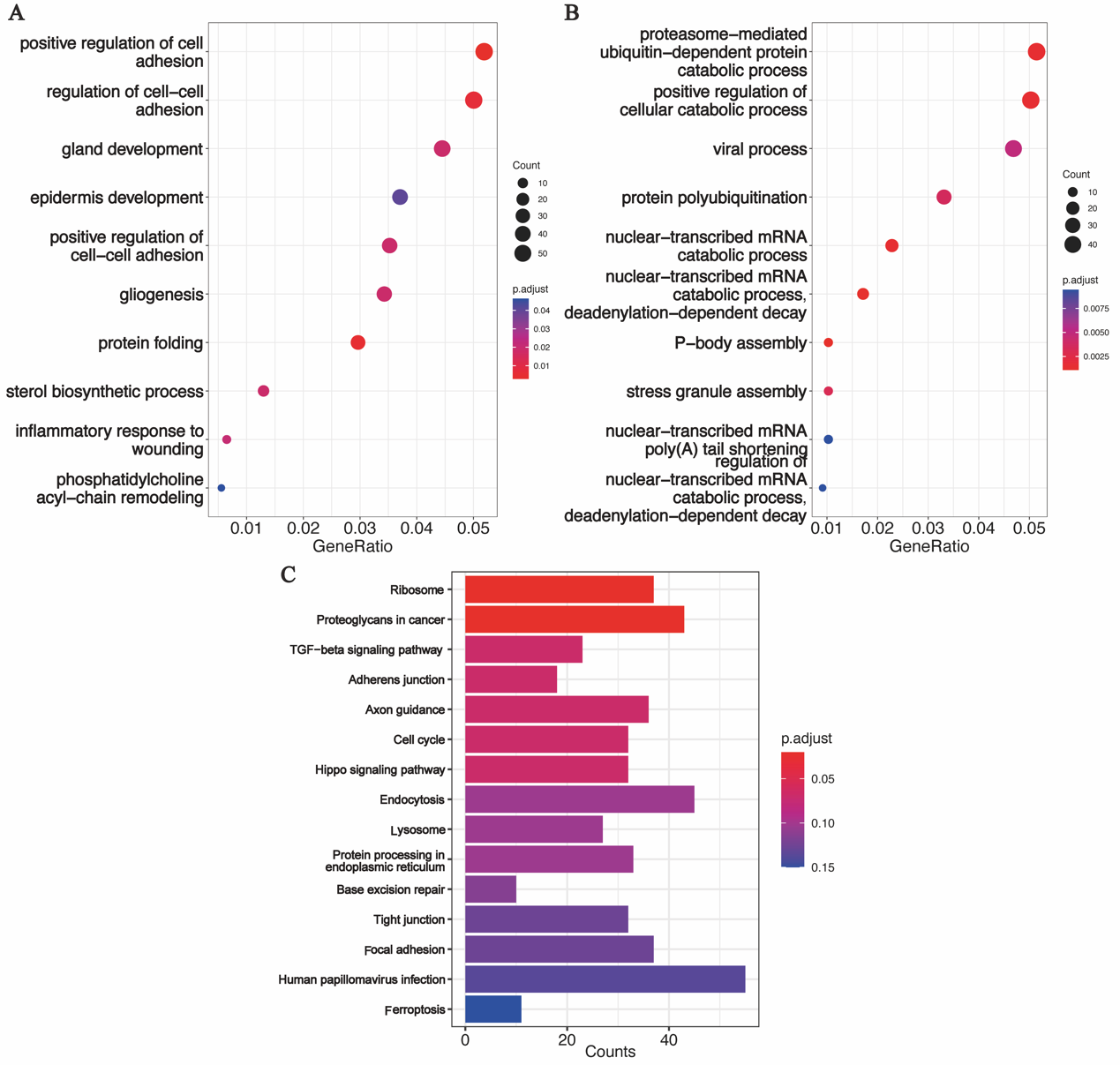


**Fig. S9** Enrichment Analysis of Biological Pathways. (**A**) GO-BP (Gene Ontology - Biological Process) enrichment analysis of upregulated DEGs. (**B**) GO-BP enrichment analysis of downregulated DEGs. (**C**) KEGG (Kyoto Encyclopedia of Genes and Genomes) pathway enrichment analysis of DEGs.
