# Supplementary material for: Clinical and Functional Characterization of CD-NTase Enzymes in Esophageal Squamous Cell Carcinoma
Source: J Cancer. 2025 Jun 12;16(9):2822–36. doi: 10.7150/jca.100226 (PMC12244066; doi:10.7150/jca.100226)
Supplement: Supplementary file 1 — Supplementary figures and tables. [file jcav16p2822s1.zip › Supplementary materials/Supplementary Table/Table S5.docx]

**Table S5 Primers used for molecular cloning of expression vectors**

| **shRNA** | **Sequence (5’-3’)** |
| --- | --- |
| Scramble shRNA | CCGGCCTAAGGTTAAGTCGCCCTCGCTCGAGCGAGGGCGACTTAACCTTAGGTTTTT |
| sh-MB21D2#1 | CCGGCTGGACTTAGATGAGCTTAATCTCGAGATTAAGCTCATCTAAGTCCAGTTTTTGAATT |
| sh-MB21D2#2 | CCGGGCTGAGCCTTCGGCTCTTTGACTCGAGTCAAAGAGCCGAAGGCTCAGCTTTTTGAATT |
| sh-MB21D2#3 | CCGGTGCAAAGCCATCATCATTAAACTCGAGTTTAATGATGATGGCTTTGCATTTTTGAATT |
| **Homo-MB21D2-NM_178496.4 5’-3’** | |
| GAATTCGCCACCAtgaagatggcggctcccaccgccaacaaggcagcctccctgggctgtaacaacaagcctgcgttcccggagctggatttcaggtcgggagctcgggtggaggaattgaacaaactcatccaagaatttacgaagcacgaccagcgggaatacgacgaccagagagcgctggagattcacacagccaaggatttcatcttttccatgctgggaatggtgcaaaagctggaccaaaagcttccagtggctaatgaatacctgttgctctctggaggtgtccgggaaggcgtggtggacctggacttagatgagcttaatgtctatgcccggggtactgactatgatatggactttaccctcttggtgccagccctcaagctgcatgaccgtaatcagcctgtgacactcgacatgcgccactcagccttgtgccactcttggctgagccttcggctctttgatgaggggacaatcagtaaatggaaagactgctgcaccattgtagatcacatcaatggtgccaccaactacttcttctcacctaccaaagtggctgactggttctatgactctatcagcattgtcctatcagaaatacagaagaaaccccagcgagggatgccaaaggtagaaaaggtggaaaaaaatgggaccatcatctccatcattctgggtgtagggagtagtcgcatgttgtatgatattgtccctgtggtatctttcaaaggttggcctgcagtggcccagagctggctcatggagaaccacttttgggatgggaagattactgaggaagaggtcatcagtgggttttacttggtgcctgcttgctcctacaagggtaagaaggacaatgaatggcggctgtcctttgccaggagcgaggtgcagttgaagaagtgcatctccagcagcctcatgcaggcctatcaggcctgcaaagccatcatcattaaactgctgtcccggcccaaggctattagcccctatcacctgcggagcatgatgctctgggcctgcgacagacttcctgccaactacttggctcaagaagactatgcagcccactttttgctgggcctcatcgatgacctgcaacactgtctggtcaacaagatgtgccccaattatttcatccctcagtgcaacatgctggaacatctgtctgaggagacagtcatgcttcacgcccggaagctgtcctctgtgcgctcagacccggcagagcacttgcgcaccgccattgagcatgtcaaggcagccaaccggctgacactggagctccagaggcgaggtagcaccaccagcatcccctctccacagtctgacggaggggaccccaaccagcctgatgaccgtttggcaaaaaaactgcagcagctagtgactgagaacccgggaaagtcaatctctgtctttatcaatcctgacgatgtcacaaggccccatttcagaattgatgacaaatttttctgaGGATCC | |
