# Supplementary material for: Clinical and Functional Characterization of CD-NTase Enzymes in Esophageal Squamous Cell Carcinoma
Source: J Cancer. 2025 Jun 12;16(9):2822–36. doi: 10.7150/jca.100226 (PMC12244066; doi:10.7150/jca.100226)
Supplement: Supplementary file 1 — Supplementary figures and tables. [file jcav16p2822s1.zip › Supplementary materials/Supplementary Table/Table S6.docx]

**Table S6 Information of antibodies used in this study**

| **Antibody** | **Company** | **Catalog number** | **Dilution** |
| --- | --- | --- | --- |
| Anti-MB21D2 | Sigma-Aldrich | HPA044026 | 1:1000 |
| Wnt3a Rabbit pAb | ZENBIPO | 822111 | 1:500 |
| Cyclin D1 Rabbit pAb | ZENBIPO | 382442 | 1:1000 |
| c-Myc Antibody | Abmart | T55150 | 1:1000 |
| Anti-Frizzled 4 | Abcam | ab277797 | 1:1000 |
| Beta-Catenin (44C6) mAb | Abmart | M24002 | 1:1000 |
| GAPDH Rabbit mAb | CST | 2118S | 1:3000 |
| β-Actin Rabbit mAb | Abclonal | AC038 | 1:3000 |
| Anti-rabbit IgG, HRP-linked Antibody | CST | 7074S | 1:5000 |
| Anti-mouse IgG HRP-linked Antibody | CST | 7076S | 1:5000 |
|  |  |  |  |
